# Measuring naturalistic speech comprehension in real time

**DOI:** 10.3758/s13428-026-02941-1

**Published:** 2026-03-27

**Authors:** Irmak Ergin, Jill Kries, Shiven Gupta, Maria Papworth Burrel, Laura Gwilliams

**Affiliations:** 1 Department of Psychology, Stanford University, Stanford, CA USA; 2 Wu Tsai Neurosciences Institute, Stanford University, Stanford, CA USA; 3 Stanford Data Science, Stanford University, Stanford, CA USA

**Keywords:** Speech comprehension, Naturalistic, Real-time measurement, Language

## Abstract

Speech comprehension has been described as an effortless and robust process; yet, in real-world contexts, it is common for a listener to misunderstand what was said or fail to derive meaning entirely. Typically, methods of measuring speech comprehension are applied ‘post hoc’ - that is, *after* the comprehension has happened. This approach fails to capture comprehension as it occurs, limiting the field’s understanding of the cognitive processes involved in real-time comprehension. To overcome these challenges, we designed and tested a novel method of measuring real-time speech comprehension during naturalistic listening. We built a slider device that synchronizes with experimental software and provides millisecond read-out. In three experiments, participants listened to audiobook segments while providing continuous comprehension ratings using the slider. To vary comprehension success, we presented speech segments at speed factors of 1–5 times faster than normal. We validated the time-resolved slider data against established speech comprehension assessment methods. Overall, our findings validate our novel time-resolved comprehension measure and demonstrate that it is possible to derive an online behavioral measure of real-time speech comprehension. We also confirmed numerous limitations of static post hoc assessments, including challenges with multiple-choice question design and the confounding of potential effects due to recency bias and comprehension for summarization. The measure proposed here overcomes the constraints of static post hoc assessments and can be effectively integrated with neuroimaging techniques, offering a valuable tool for future research on dynamic processes during naturalistic listening.

**Supplementary Information:**

The online version contains supplementary material available at 10.3758/s13428-026-02941-1.

## Introduction

How the human brain implements speech comprehension is a foundational question that cuts across cognitive science, neuroscience, and computer science (Adolfi, Bowers, & Poeppel, 2023; Gwilliams, 2020; Gwilliams et al., 2024; Mehrish, Majumder, Bharadwaj, Mihalcea, & Poria, 2023; Meyer, 2018; Specht, 2014). Comprehension is an internally derived state, which is achieved rapidly in real-time and can fluctuate over the course of an interaction (Davis and Johnsrude, 2003; Peelle, McMillan, Moore, Grossman, & Wingfield, 2004; Weissbart, Kandylaki, & Reichenbach, 2020). The ability to measure comprehension in real-time, and identify the conditions under which comprehension succeeds or fails, is a critical precursor for understanding how the system operates (Mattys, Davis, Bradlow, & Scott, 2023). Ultimately, linking subjectively perceived real-time comprehension to neural activity will provide important insight into the neural implementation of the system (Poeppel, Idsardi, & Van Wassenhove, 2008; Rodd and Davis, 2017).

**Fig. 1:**
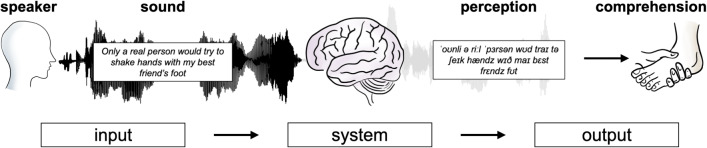
Speech perception and speech comprehension. Speech perception refers to sub-lexical operations, and it is necessary for comprehension. Yet, it does not always predict or correlate with the ability to construct representations from what has been heard, that is comprehension

While speech comprehension is often described as a robust and automatic process (Gwilliams et al., 2024; Gwilliams and Davis, 2022; Gwilliams, King, Marantz, & Poeppel, 2022; Shannon, Zeng, Kamath, Wygonski, & Ekelid, 1995), it is common for a neurotypical adult listener to misunderstand, or to fail to derive meaning entirely. This can happen for a number of reasons. One relates to a failure of the perceptual system, whereby the listener fails to hear and parse the speech input appropriately (Hickok & Poeppel, 2000). This might be due to degraded acoustic input (Davis, Johnsrude, Hervais-Adelman, Taylor, & McGettigan, 2005; Hervais-Adelman, Davis, Johnsrude, & Carlyon, 2008), increased environmental noise, or competing voices (Aydelott, Baer-Henney, Trzaskowski, Leech, & Dick, 2012; Bronkhorst, 2000; McDermott, 2009). A second factor concerns the comprehension system proper (Fig. 1). In this case, a listener may be able to perceive the sounds but fail to extract the intended meaning. This is likely to happen under the condition of high information load (Gwilliams & Davis, 2022); Koskinen, Kurimo, Gross, Hyvärinen, & Hari, 2020; Savin, 1963), or speech rate (Griffiths, 1990; Kuperman, Kyröläinen, Porretta, Brysbaert, & Yang, 2021; Lubinus, Keitel, Obleser, Poeppel, & Rimmele, 2023; Vagharchakian, Dehaene-Lambertz, Pallier, & Dehaene, 2012; Verschueren, Gillis, Decruy, Vanthornhout, & Francart, 2022). Finally, external cognitive processes such as attention can serve to limit speech comprehension (Rimmele, Golumbic, Schröger, & Poeppel, 2015; Wild et al., 2012).

Given that comprehension is a primarily internal experience, it is a challenging cognitive state to measure (Hickok & Poeppel, 2007). Prior research has primarily used ‘post hoc’ assessments, which test a listener’s comprehension *after* the comprehension has happened. For example, the listener may be instructed to answer a multiple-choice question about the speech content they just heard, with the logic that if they did not comprehend, they will not be able to answer the questions accurately. This approach is used both in experimental contexts (Heilbron, Armeni, Schoffelen, Hagoort, & De Lange, 2022; Kries et al., 2024; Moody, Joost, & Rodman, 1987; Wester, Watts, & Henter, 2016) and for language skill evaluation purposes, such as the Test of English as a Foreign Language (TOEFL), and IELTS (International English Language Testing System). Another post hoc method is to ask listeners to estimate how much they understood on a scale (Gillis, Vanthornhout, & Francart, 2023; Schiavetti, 1992), which requires participants to introspect on their own internal experience of comprehension and answer truthfully. Finally, ‘word identification tasks’ ask listeners to repeat the words or sentences they heard as accurately as possible, and the number of true recalls constitutes the comprehension score (Beukelman & Yorkston, 1979; Davis et al., 2005; Lubinus et al., 2023).

These post hoc measurements suffer from three main limitations in measuring speech comprehension. (1) Responses given after stimulus processing rely on memory. This means that these measures capture not only how well the listener understood the spoken language, but also how well they remembered it. Indeed, working memory is hypothesized to play a critical role in speech comprehension, and previous studies have shown that working memory capacity predicts performance in continuous speech comprehension when measured with post hoc tasks (Emmorey, Giezen, Petrich, Spurgeon, & Farnady, 2017; Lubinus et al., 2023; Tun, Wingfield, & Stine, 1991). (2) Numerous neuroimaging studies have recorded neural responses during speech listening and related them to comprehension performance (e.g., Heilbron et al., 2022; Kries et al., 2024; Gillis et al., 2023). However, these links are made with a post hoc measure that yields a single-value score, which does not match the temporal resolution of neural responses or the underlying speech processes. Because speech is a dynamic input, listeners’ comprehension fluctuates over time: some moments are understood well, others poorly. This granularity mismatch limits a direct, time-locked correspondence between fluctuations in the neural signal and fluctuations in comprehension. (3) Measures like multiple-choice questions are difficult to evaluate on a single-trial basis, as subjects have a chance of answering correctly without truly understanding the input. Questions are often chosen arbitrarily, cannot cover the full content, and their formulation may allow subjects to infer the correct answer from context or rely on general word knowledge.

With these limitations in mind, the primary objectives of the present study are to (1) test the nature and extent of the limitations of post hoc comprehension measures; (2) develop and validate a method of recording speech comprehension in real time; which can (3) be utilized simultaneously with neuroimaging recordings.

To achieve these objectives, we built a paramagnetic slider device that allows participants to report continuous comprehension ratings while listening to naturalistic continuous speech. In Experiment 1, we performed a speeded listening task, in which participants listened to audiobook segments that are easy to understand (x1), somewhat challenging to understand (x2, x3), and very challenging to understand (x4, x5). Using this paradigm, we validate our self-report measure by comparing it to the post hoc comprehension assessments that have been used in prior literature, described above. This consists of a 10-point scale rating, a written summary, and a four-alternative multiple-choice question after each speech segment. In addition to comprehension measures, we also measured participants’ working memory capacity and auditory acuity. This allowed us to account for the variability in comprehension ratings that are not due to comprehension per se, but rather related to working memory limitations or a general performance deficiency in speech perception (Billings, Olsen, Charney, Madsen, & Holmes, 2023; Taylor, 2003; Skoe and Karayanidi, 2019).

In Experiment 2, we examined whether the slider could be applied in longer, continuous listening settings suitable for neuroimaging studies of naturalistic listening paradigms. Participants listened to 10-min audio segments in which speech rate (x1–x5) was manipulated continuously, while providing real-time comprehension scores using the slider.

Finally, in Experiment 3, we test whether the slider can be used to track multiple comprehension challenges. Specifically, we manipulated information load by including high- versus low-surprisal moments in the story, as well as slow and fast versions of the segments. We then leveraged the time-resolved nature of the slider by modeling response delays for individual participants, showing that these delays can be characterized and incorporated into future neuroimaging studies.

The results of our three experiments demonstrate empirically that our slider device provides a real-time measure of a listener’s speech comprehension. The ability to record language comprehension in real time will provide speech scientists with critical, and previously inaccessible, insight into the causal neural mechanisms underlying speech comprehension by directly relating comprehension success on a moment-by-moment basis with neural encoding of sensory and symbolic information in future studies.

## Experiment 1

### Methods

#### Participants

Thirty native English speakers without any diagnosed hearing or neurological disorders participated in the study (12 females, *mean age*= 25.27 years, *age range*= 18–46 years). They received course credit or monetary compensation for their participation. We eliminated eight participants’ data because they failed the preregistered multiple-choice question accuracy criterion (see Supplementary Materials for details).

#### Materials

##### Stimuli

An audiobook, *Someday, Someday, Maybe* (Graham, 2014), served as the source for our original recording, from which we extracted segments that were approximately 30 s long, and started and ended with sentence beginnings and endings. These segments were sped up to create stimuli at five different speech rates, ranging from a minimum speeding-up factor of 1 (the same as the original recording rate) to a maximum factor of 5 (five times the original recording rate). The speech rate conditions are composed of the original recording rate, and 2, 3, 4, and 5 times faster than the original recording rate. Segments were compressed in Praat (Boersma & Weenink, 2024) by a given factor while keeping the pitch unchanged. We used the Praat Vocal Toolkit plugin, from which we used the function *changevtpitchduration.praat*. We slightly customized the function by setting the variables new_dur and original_dur to correspond to: $\text {segment duration} \times \left( \frac{1}{\text {speeding up factor}}\right)$. This allowed us to speed up each segment by a factor rather than to a fixed duration, and thus to maintain consistent speech rates, avoiding variations caused by small differences in original segment lengths. The mean durations of audio segments were 29 s (*SD* = 1.33) for speech rate x1, 14.55 s (*SD* = 0.57) for speech rate x2, 10.93 s (*SD* = 0.31) for speech rate x3, 7.28 s (*SD* = 0.26) for speech rate x4, and 5.81 s (*SD* = 0.26) for speech rate x5. This corresponds to an average of 195.76 words per minute (wpm) for speech rate x1, 389.46 wpm for speech rate x2, 525.49 wpm for speech rate x3, 779.70 wpm for speech rate x4, and 978.89 wpm for speech rate x5. In total, we used 125 segments, consisting of 25 instances for each of the five speech rates. We randomized segment-to-speed allocation across participants to avoid potential differences in context or word salience, and to ensure uniformity in the difficulty of post hoc comprehension questions.

### Measures

#### Control measures

##### Digit Span

We measured working memory capacity using the forward and backward Digit Span Test (Richardson, 2007; Wechsler, 1981). The forward and backward tasks consisted of different digit sets, and the forward test was applied before the backward test. Each of them included seven levels (3–9 digits) with two different spans (sequences) for each length level. The procedure started with the shortest digit spans (three digits) and stopped as soon as participants failed to correctly repeat both spans belonging to one level or when the two longest digit spans (nine digits) were successfully completed. Digit spans were presented auditorily, and participants responded verbally.

##### Digit-In-Noise Test

We measured auditory acuity using (“hearWho”) - a mobile and web-based software application for hearing screening developed by the World Health Organization (WHO). HearWho utilizes a Digit-In-Noise Test by presenting 23 spoken sets of US English digit triplets over progressively increasing white noise and provides a percentage score of auditory acuity (WHO, 2022). Participants were instructed to adjust the volume to a comfortable level before they started the task.

### Speech comprehension measures

#### Real-time speech comprehension measure

Participants reported continuous comprehension on a custom-built physical slider while listening to the segments. They expressed their comprehension level by adjusting the distance between their index finger and thumb (Pelli & Vale, 2014) — maximizing the distance indicates complete comprehension (a maximum value of 255) while minimizing signifies no comprehension (a minimum value of 0). This reporting approach allows participants to convey continuous feedback without having to visually engage with the slider, relying on their sense of distance between their index finger and thumb of the same hand. Accordingly, our approach differs from common screen-based reporting tools such as marking comprehension on a spectrum using mouse tracking or touchscreen interfaces (Apfelbaum, Kutlu, McMurray, & Kapnoula, 2022; Kutlu, Chiu, & McMurray, 2022) in two key respects: (1) screen-based methods typically require visual guidance, which is undesirable in neuroimaging contexts because it can induce head-movement artifacts and complicate co-registration to neural data; by contrast, the slider can be operated by using the perceived distance between the thumb and index finger, with fixed end-stops that provide clear min/max bounds; and (2) the device is constructed to be paramagnetic to enable compatibility with neuroimaging methods such as electroencephalography (EEG) and magnetoencephalography (MEG), thereby facilitating potential follow-up studies (see “Discussion” section).

The slider is a standalone device built around an ARM-Cortex M0 that can be plugged into the data acquisition computer, and it has two main components: [1] a microcontroller development board, and [2] a slide potentiometer. The slider provides a readout every 4 ms for a sample rate of 250 Hz, which corresponds to approximately 7250 samples for speech rate x1, 3638 for speech rate x2, 2733 for speech rate x3, 1820 for speech rate x4, and 1453 for speech rate x5. For simplicity, we chose the Adafruit Feather M0 as the microcontroller and a 100-mm, 10-kOhm slide potentiometer. Below, we outline the rationale behind these choices.

1. *Microcontroller unit (MCU):* The MCU serves as the interface between the slider and the experiment computer, communicating over a USB connection. For this to work, the chip must either support native USB communication or use an additional converter chip (such as FTDI) to send data via a serial protocol (Universal Asynchronous Receiver-Transmitter (UART)). We configured the analog-to-digital converter (ADC) to produce 8-bit readings, values ranging from 0 to 255, since we prefer less noise and do not need a higher resolution.
2. *The slide potentiometer:* The three main considerations for the slider were travel length, tolerance (how much the actual resistance could deviate from the nominal 10k$\Omega$), and electrical noise—such as fluctuations in the voltage signal not caused by the user’s movement. A 100-mm travel length provides a comfortable range for participants to make smooth, continuous responses. Although the slider has a 20% tolerance, this is not a major concern, as any electrical noise or resistance fluctuations during movement can be assessed using an oscilloscope. In our testing, we did not observe any significant electrical instability. Since participants are unlikely to perceive minor positional shifts on the order of a few millimeters, such small variations are not expected to affect the experiment.

**Fig. 2:**
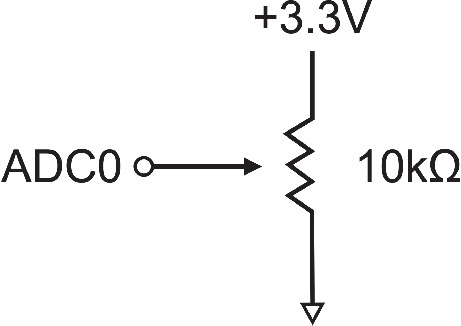
The circuit diagram

The circuit diagram for the device is as follows: One end of the potentiometer is connected to the MCU’s power line, the other to ground, and the output is sent to one of the MCU’s ADC pins (Fig. 2).

**Fig. 3:**
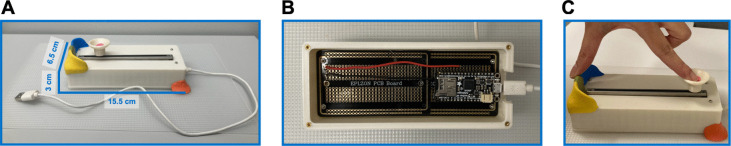
**A.** Slider device. Dimensions: $15.5\,\text {cm} \times 3\,\text {cm} \times 6.5\,\text {cm}$. **B.** Open enclosure of the device. **C.** Demonstration of hand placement on the device. A demo video is available at https://github.com/irmak-ergin/measuring_naturalistic_speech_comprehension_2024/blob/main/experiment_1/writeup/figures/demo.mp4

##### Device construction

The slider and microcontroller are soldered onto a protoboard (Fig. 3B). Please note that poor protoboard quality can introduce electrical noise. Next, we designed a 3D printed case to protect the circuitry and provide a better experience for the participant. For durability, we used heated inserts so the device can be disassembled without damaging the plastic parts. Finally, we mounted a plastic knob on top to help reduce fatigue during use (Fig. 3A).

##### Software

To integrate the device into our experiment software (PsychoPy), we configured the MCU to send data continuously over USB. This enables the internal clock to be synchronized with PsychoPy, which is in turn synchronized with the audio presentation. Each transmitted data point includes both a slider reading and a precise timestamp. This is necessary because USB communication can introduce unpredictable timing delays, and the MCU and computer clocks are not perfectly synchronized, a phenomenon known as clock drift.

The computer logs each incoming data point during the experiment. Since the computer clock is aligned with the audio playback timeline, it serves as the reference (“golden”) clock. We estimate the clock drift by comparing elapsed time on the MCU and the computer. Our tests show that the drift remains below 0.05% over a trial- well below thresholds that would affect behavioral measurements. Note any delay between the time PsychoPy reports the audio file was started, versus when the computer driver starts playing the sound, was not measured, as it sounds instantaneous to any subject. You can find the details about the MCU and PsychoPy code in the Supplementary Materials.

#### Post-hoc comprehension measures

##### 10-point scale response

After each segment is finished, participants rated their comprehension on a ten-point scale, with 0 indicating no comprehension and 10 indicating full comprehension. This task enabled us to have a post hoc self-report comprehension measure on a continuous scale similar to the real-time comprehension measure.

**Fig. 4:**
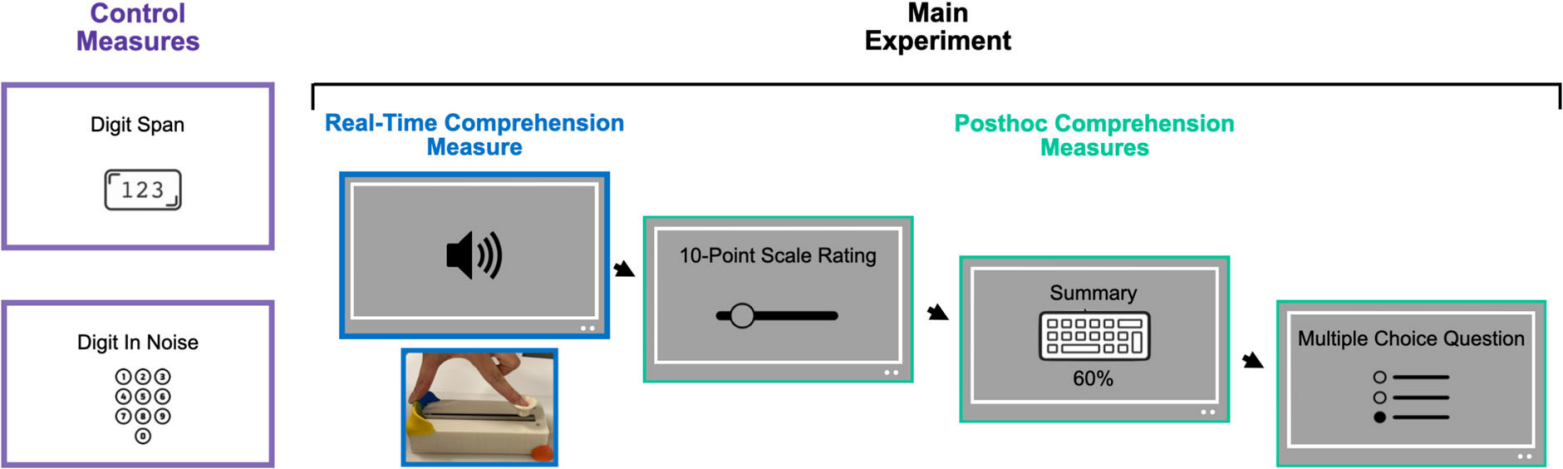
Experiment 1 paradigm. Participants did the Digit Span and Digit In Noise tests before the main experiment. The experiment started with two training blocks, the first one with the slowest and the second one with the fastest speech rates, before 125 experimental blocks. Each block started with a speech segment randomly presented in one of five speech rates. Participants reported their real-time comprehension using the slider while listening to the segment. After listening, they completed the post hoc comprehension tasks. The summary task was present only for 60% of the blocks

##### Summary task

Participants had a maximum of 30 s to provide a written summary for the segments. Summaries were required for 60% of the trials only, to ensure the experiment duration did not exceed 2 h. We evaluated the semantic similarity of participants’ summaries to the speech segments with three different calculation methods using two different word embedding models to account for how different models might elucidate the behavioral data differently. (1) Using GLoVe (Pennington, Socher, & Manning, 2014), the cosine similarity of every word in the written summary and the text of the audio segment of each trial is calculated. Then, the maximum cosine values for each summary word were summed to have a semantic summary score that is sensitive to the word count, as well as the similarity of each word (referred to as GloVe- Written Summary). This prevents summaries with a single exact word match with the segment from having the highest similarity value, while ensuring longer conceptual summaries that do not match verbatim are not unfairly penalized. (2) Using GLoVe, the cosine similarity of every word in the text of the audio segment and the summary of each trial is calculated. Obtaining cosine values for speech segment words rather than summary words is another way of preventing rewarding a few perfectly matched words, as segments have a similar number of words. Then, we calculated the average of the maximum cosine values of each audio segment word to obtain the semantic similarity score (referred to as GloVe- Heard Segment). These two methods using GloVe provide a word-based comparison while overcoming the binary assessment problem (‘cat’ will have a different score than ‘rain’ when compared with ‘dog’). Yet, GloVe word embeddings cannot take semantic context into account (for instance, it cannot differentiate between “cat under the table” and “table under the cat”) which might have an impact on the semantic similarity assessment of the whole summary; (3) Using BERT (Reimers & Gurevych, 2019), which is context sensitive, we calculated the cosine semantic similarity. This enabled us to obtain a single semantic similarity score for the summary compared to the speech segment text.

**Fig. 5:**
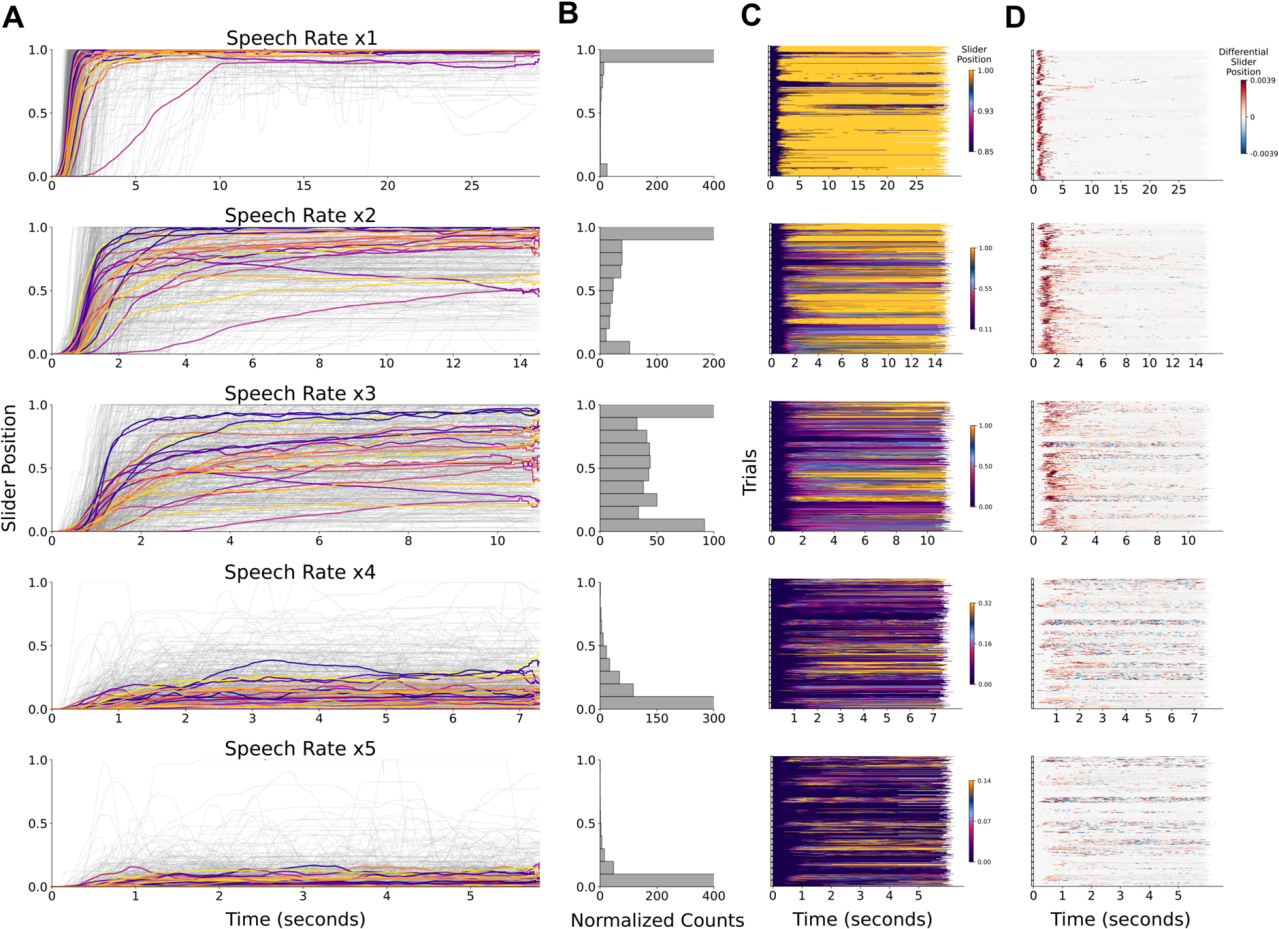
Slider responses by speech rate. **A:** Normalized time-resolved slider scores reported via the slider during each trial for each speech rate. *Grey lines* represent individual trials, while *colored lines* show the average slider values across trials for each participant at each speech rate. The *x*-axis represents time in seconds, and the *y*-axis represents the slider position. **B:** The histograms depict how many times (*x*-axis) each value reported via the slider appeared across trials for each speech rate. The counts are normalized by dividing the number of values by the mean duration of the respective speech rate’s trials for visualization purposes. The values (*y*-axis) are grouped into ten bins. **C:** The slider position is plotted over each trial’s time course (*x*-axis). Each bracket marks the transition between participants, with each row representing a trial. The color scale’s minimum and maximum values are set to the 10th and 90th percentiles of slider positions for each speech rate condition. **D:** The differential of slider position is plotted over each trial’s time course (*x*-axis). The differential at each time point is the change between consecutive slider values. Each *bracket* marks the transition between participants, with each *row* representing a trial. The color scale’s minimum and maximum values are set to the 1st and 99th percentiles of slider positions for each speech rate condition

##### Multiple-choice questions

We initially tried using generative AI (ChatGPT 3.5) (Vaswani, 2017) to generate four-option multiple choice questions by providing the transcript of each audio segment. This approach, however, did not yield conceptually clear questions with equally challenging options and a single, distinctly correct answer. Consequently, as is the typical approach in the field, we opted to write the questions ourselves (Gwilliams et al., 2023a; Heilbron et al., 2022; Wester et al., 2016; Zekveld, Heslenfeld, Festen, & Schoonhoven, 2006). In doing so, we aimed to keep the questions equally challenging and tried to draw the content of each question from varied time points across segments. The multiple-choice accuracy score was calculated by assigning a score of 1 to correctly answered questions and 0 to incorrectly answered ones.

##### Design and procedure

Firstly, participants completed the control tasks, which included the working memory task using Digit Span, and the speech perception in noise task using the Digit In Noise test. Then, prior to the experimental trials, participants had two training blocks with the slowest and fastest speech rates, respectively, to get familiar with the range of speech rate conditions and the protocol. The experiment employed a block design. Each of the 125 blocks started with listening to a speech segment while simultaneously rating speech comprehension using the slider and ended with post hoc comprehension measures, i.e., a ten-point scale comprehension rating, a summary, and a multiple-choice question. The multiple-choice questions were placed last to avoid providing participants with information that could influence their summaries or scale ratings. Each block consisted of a presentation of a single speech rate. The 125 blocks were randomly ordered (Fig. 4). The experiment was administered via PsychoPy v.2023.2.3 (Peirce et al., 2019).

#### Analysis

We rescaled all variables within a 0–1 range before applying our analyses to make the model coefficients comparable. We used Python version 3.11.8 (Van Rossum & Drake, 2009) to conduct the semantic similarity analysis and fit linear and sigmoidal models to slider data, and RStudio version 2023.12.1+402 (Posit Team, 2024) for all other analyses. All analyses were preregistered except the recency analysis and the investigation of the nature of decline in comprehension (linear vs. categorical), which were exploratory.

### Results

We took the median of the time course of slider values to derive a single comprehension score for each trial. In each regression model, we included the digit span score and digit in noise scores as random slopes to account for by-subject variability in comprehension scores that are not due to comprehension per se. We used the lme4 R package (Bates, Mächler, Bolker, & Walker, 2015) for all regression analysis.

#### Slider scores

To test whether comprehension declines with increasing speech rate, we used a linear mixed effects regression model. This includes a fixed effect for by-trial speech rate, and by-subject random slopes for working memory and auditory acuity abilities. The outcome variable was the median slider score obtained through the slider device. We implemented this analysis using the following syntax:

$$\begin{aligned} \texttt{lmer}&\Big (\mathtt {median\_slider\_score} \sim \mathtt {speech\_rate}\\&+ (1 \mid \mathtt {digit\_span\_score}) \\&+ (1 \mid \mathtt {digit\_in\_noise\_score}) \Big ) \end{aligned}$$

Speech rate was a significant predictor of the median slider values. In line with our hypothesis, as speech rate increased, slider scores decreased ($\beta =-0.26$, ${SE}=0.003$, ${t}(2733)=-102.27$, *p*<.001). Figure 5 shows time-resolved slider scores reported via the slider during each trial, and participant averages across trials for each speech rate, obtained by taking the mean of slider scores reported for each time point across trials. Despite the individual variability in comprehension reports, all participants reported comprehending less with increasing speech rate. Moreover, participants used the slider dynamically during listening, as evidenced by the fluctuations observed in individual trials, showing that they are actively engaging with the slider over time.

#### Quantifying summary performance

Next, we validated our time-resolved measure against traditional post hoc measures. For the written summaries, we aimed to numerically represent their semantic content using word embeddings and compare their semantic similarity to the semantic content of the heard passages.

Because there are a few different ways of performing a semantic similarity analysis, we first tested different approaches to assess the optimal method. BERT (Reimers & Gurevych, 2019) is a context-sensitive language model that can be used to provide a single vector to represent a language segment. GloVe (Pennington et al., 2014) is a context-free word embedding dictionary, formed from word co-occurrences. Because GloVe provides a vector for each word, rather than a vector for each segment, we can compute the semantic similarity using GloVe in two different ways. One way is to take each word in the summary and compare it to each word in the audio segment, then take the maximum cosine value for each summary word, and sum these maximum values across all words to obtain one score for that summary (GloVe–Written Summary). Another way is to compare each word in the audio segment with each word in the summary instead, and calculate the average of the maximum cosine values of each audio segment word (GloVe–Heard Segment). Thus, this provides us with three semantic similarity measures: BERT, GloVe-Written Summary, and GloVe-Heard Segment (You can find a more detailed account of each semantic similarity measure in the Measures section.).

First, we computed the Pearson correlation between three semantic similarity measures to examine their relationship. The correlation between BERT and GloVe- Written Summary was positive and moderate (${r}= 0.54$, ${t}(1482)=24.63$, *p*<.001). The correlation between BERT and GloVe- Heard Segment was positive and moderate (${r}=0.57$, ${t}(1482)=26.71$, *p*<.001). Finally, the strongest correlation was observed between the two GloVe-based measures (${r}=0.78$, ${t}(1482)=48.07$, *p*<.001). Then, we used the ten-point scale comprehension rating provided at the end of the passage to determine which of the three approaches was optimal, so that we do not ‘over-fit’ the approach for the slider responses we aim to validate. We performed a linear mixed effects regression analysis to examine how each semantic similarity measure as a fixed effect predicts comprehension, measured by post hoc ten-point scale rating responses, while accounting for individual differences in working memory and digit in noise capacities as random slopes over subjects. All three methods were significant predictors of comprehension, but GloVe-Written Summary semantic similarity scores explained the most variance (BERT: $\beta =0.48$, ${SE}=0.04$, ${t}(1472)=11.34$, *p*<.001; GloVe-Heard Segment: $\beta =0.28$, ${SE}=0.08$, ${t}(1465)=3.57$, *p*<.001; GloVe-Written Summary: $\beta =0.83$, ${SE}=0.04$, ${t}(1473)=18.55$, *p*<.001). Therefore, we used the GloVe-Written Summary semantic similarity scores in the rest of the analysis.

#### Validating the slider measure

To validate our time-resolved measure against traditional post hoc measures, we used mixed effects linear regression with median slider responses across time as the independent variable, and three post hoc measures as fixed effects. This method evaluates the extent to which the slider scores co-vary with post hoc comprehension scores. Firstly, separate regression models assessed each post hoc test’s ability to predict median slider scores, using the R syntax below:

$$\begin{aligned} \texttt{lmer}&\Big ( \mathtt {median\_slider\_score} \sim \mathtt {10\_point\_scale}\\&+ (1 \mid \mathtt {digit\_span\_score}) \\&+ (1 \mid \mathtt {digit\_in\_noise\_score}) \Big )\\ \texttt{lmer}&\Big ( \mathtt {median\_slider\_score} \sim \mathtt {semantic\_summary}\\&+ (1 \mid \mathtt {digit\_span\_score}) \\&+ (1 \mid \mathtt {digit\_in\_noise\_score}) \Big )\\ \texttt{lmer}&\Big ( \mathtt {median\_slider\_score} \sim \mathtt {multiple\_choice\_accuracy}\\&+ (1 \mid \mathtt {digit\_span\_score}) \\&+ (1 \mid \mathtt {digit\_in\_noise\_score}) \Big ) \end{aligned}$$

Secondly, we modeled the slider scores with all three post hoc measures as fixed effects:

$$\begin{aligned} \texttt{lmer}&\Big (\mathtt {median\_slider\_score \sim 10\_point\_scale}\\&\mathtt {+ semantic\_summary + multiple\_choice\_accuracy}\\&\mathtt {+ (1 \mid digit\_span\_score) + (1 \mid digit\_in\_noise\_score)}\Big ) \end{aligned}$$

This allowed us to examine whether the post hoc comprehension measures could explain independent variance in slider scores and to consider the variation in how each measure captures different aspects of comprehension.

**Fig. 6:**
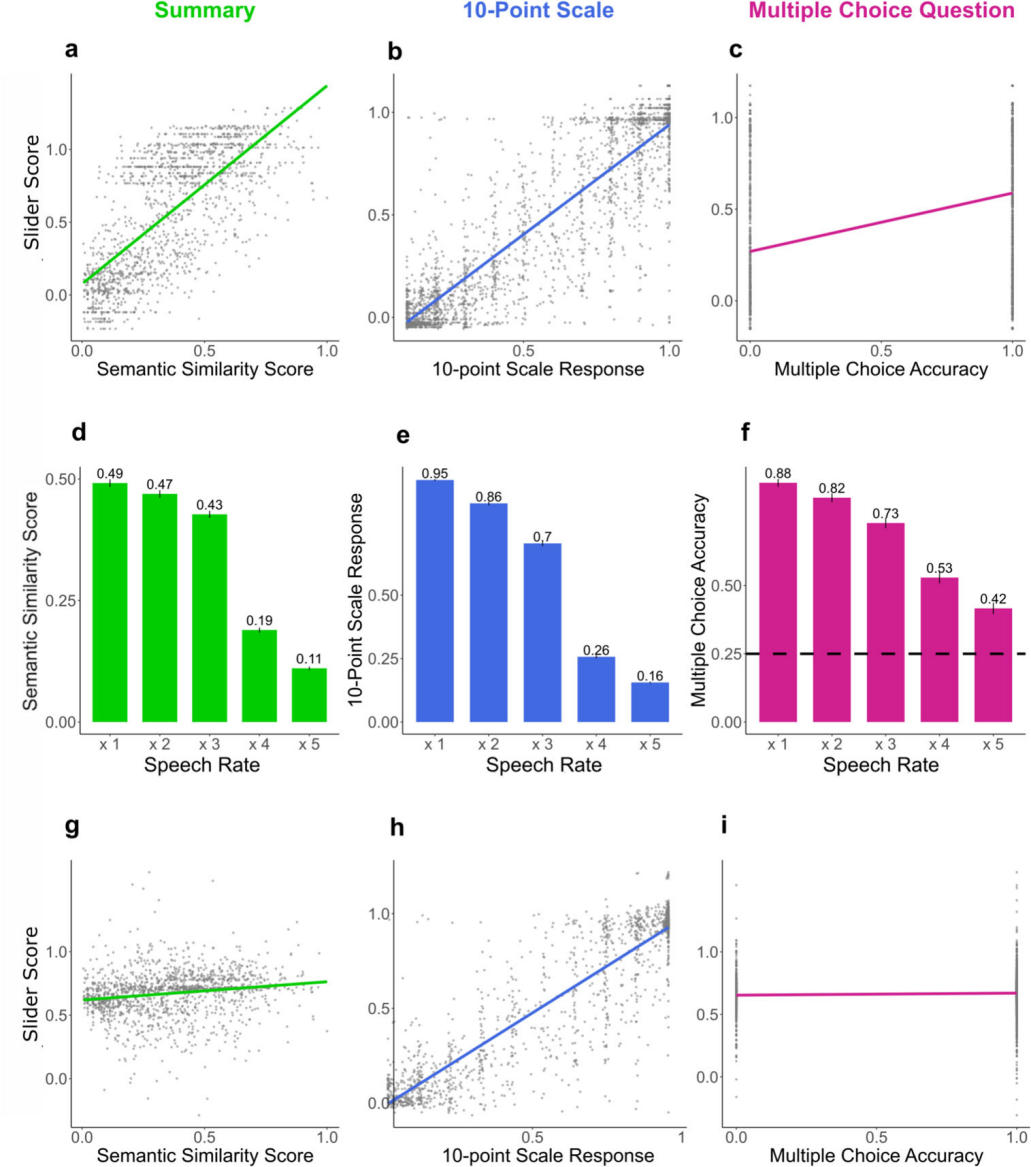
*Top row* Regression plots predicting slider scores from each post hoc comprehension score. All post hoc comprehension scores were significant predictors of slider scores when utilized as the only fixed effect. Each plot shows the relationship between the specified post hoc comprehension measure (*x*-axis) as the predictor and the predicted slider score (*y*-axis). *Middle row* Mean scores per speech rate for each post hoc comprehension measure. *Error bars* represent standard error. The *dashed line* in panel f represents the chance level. *Bottom row* Regression predicting slider scores from all post hoc comprehension scores as fixed effects. Each *plot* shows the relationship between the specified post hoc comprehension measure (*x*-axis) as the predictor and the predicted slider score (*y*-axis), holding the other predictors constant at their mean values. Semantic similarity scores and ten-point scale ratings were significant predictors, whereas multiple-choice question accuracy was not

All three mixed effects linear regression analyses predicting slider scores from each post hoc comprehension scores as a single fixed-effect were significant (ten-point scale: $\beta = 1.07$, ${SE}= 0.01$, ${t}(2736) = 109.88$, *p* <.001; semantic similarity: $\beta = 1.36$, ${SE}= 0.04$, ${t}(1479) = 36.64$, *p* < .001; multiple-choice accuracy: $\beta = 0.32$, ${SE}= 0.02$, ${t}(2741) = 19.94$, *p* < .001; Fig. 6, top row).

When we conducted the mixed effects linear regression analyses with all three post hoc measures as fixed effects, we observed a significant effect of ten-point scale rating and semantic similarity of the summaries ($\beta = 0.14$, ${SE}= 0.03$, ${t}(295.32) = 4.47$, *p* <.001 and $\beta = 1.03$, ${SE} = 0.02$, ${t}(403.09) = 50.14$, *p*<.001, respectively), and non-significant trend for multiple-choice accuracy ($\beta = 0.02$, ${SE}= 0.01$, ${t}(1468.56) = 1.49$, ${p}=0.14$) (Fig. 6* bottom row*). These results suggest that our novel measurement assesses comprehension as intended.

**Fig. 7:**
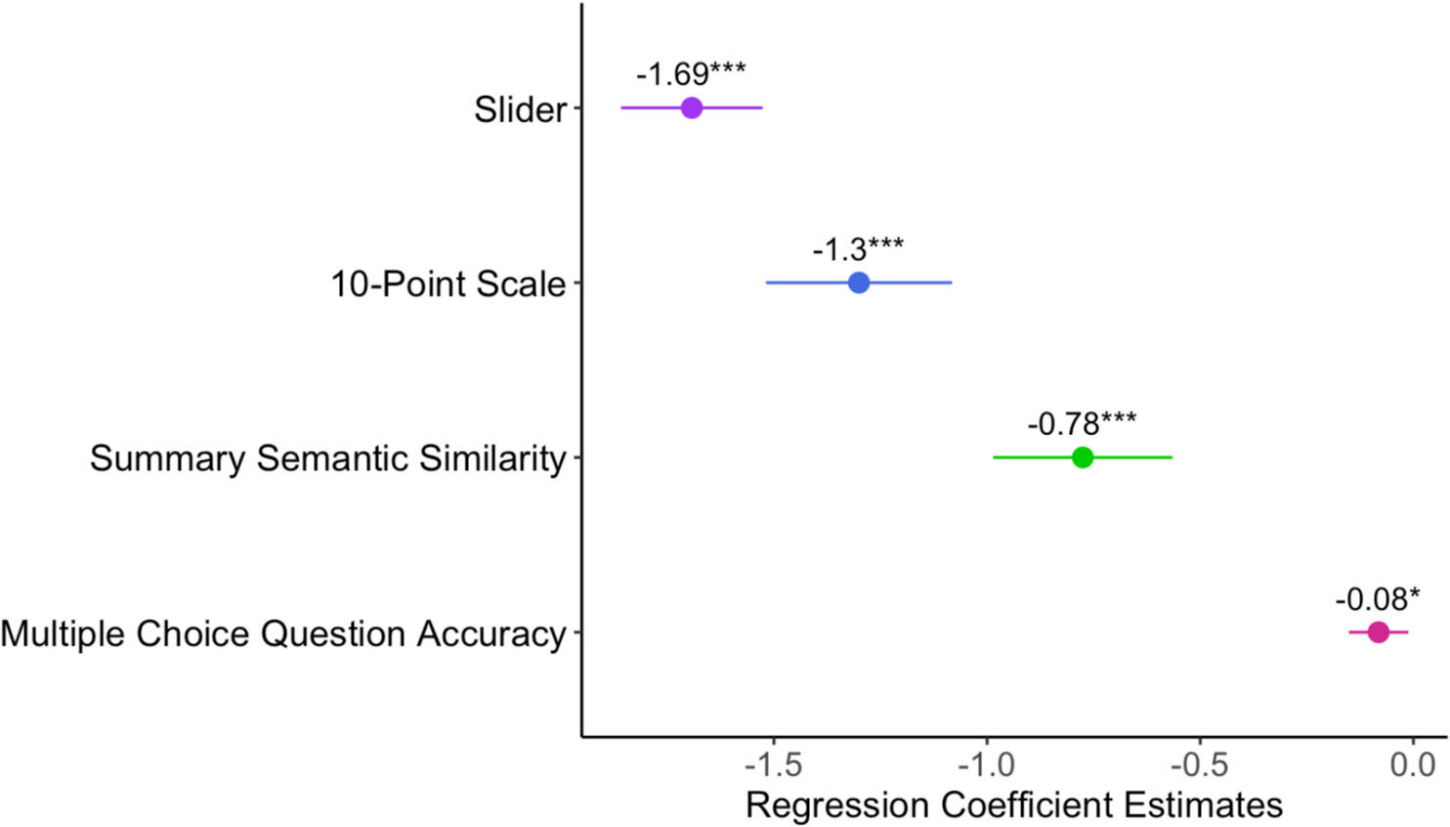
Predicting speech rate from comprehension scores. Regression coefficient estimates of the comprehension scores predicting speech rate. *Horizontal lines* represent 95% confidence intervals.***= p<.001, **= p<.01, *= p<.05

Furthermore, we found that all comprehension measures significantly predicted speech rate, such that as speech rate increased, comprehension scores decreased. Out of all the measures, the slider was the predictor with the largest beta coefficient (Fig. 7).

**Fig. 8:**
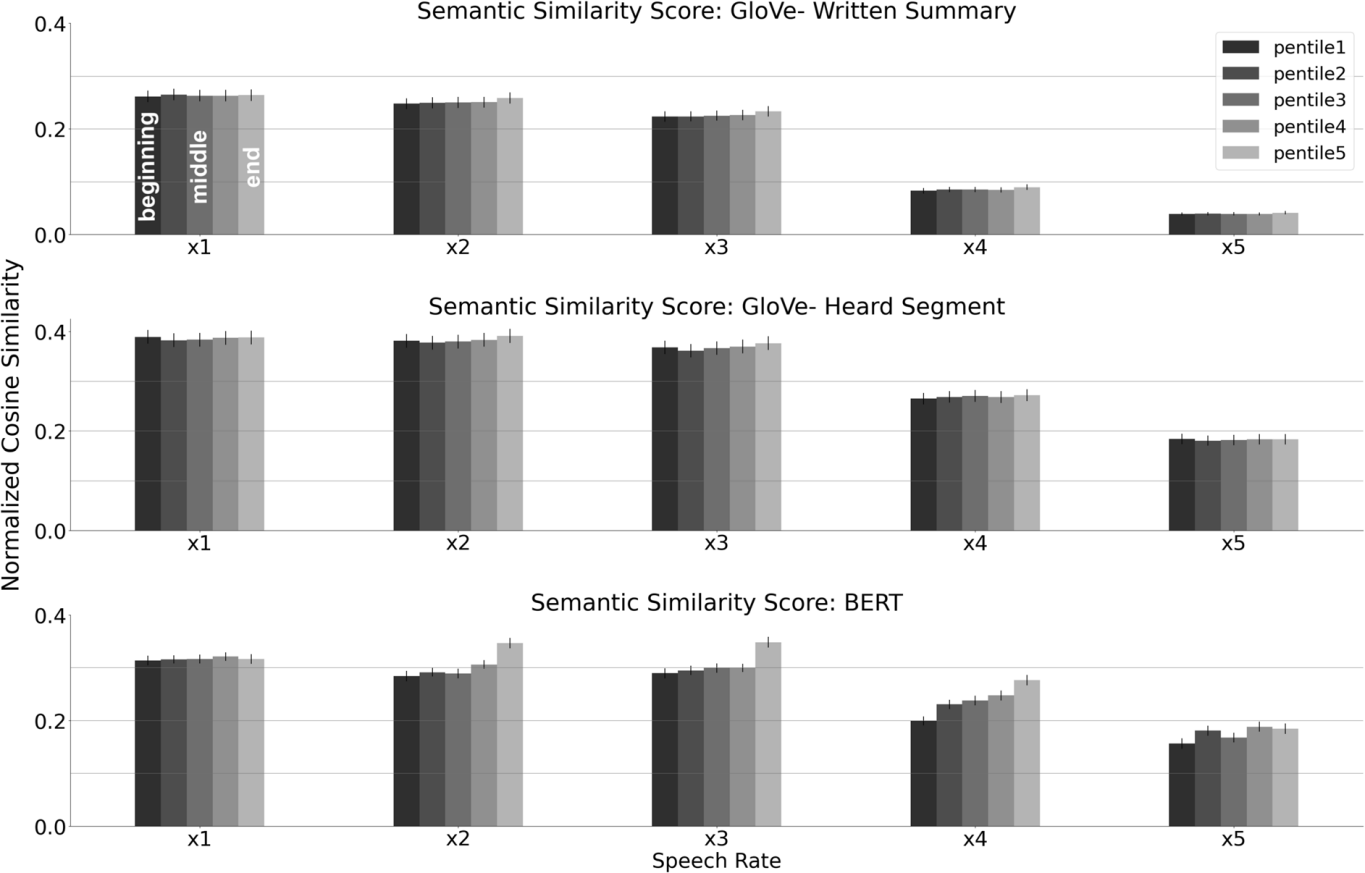
Semantic similarity between written summaries and speech segment parts. To examine possible memory effects on participants’ summaries, we divided the audio segment texts into five bins (pentiles) and calculated the semantic similarity between the summary and each pentile. Semantic similarity calculation methods can be found in the Measures section. *Error bars* represent standard errors

The variance explained by the by-subject variables, i.e., working memory and auditory acuity, was negligible in all regression analyses.

#### Investigating memory effects in summaries

To examine potential memory effects on participants’ written summaries, we tested whether participants disproportionately wrote about certain parts of the segments more than others – e.g., were biased to write more about the end of the segments rather than the middle. We divided the speech segment texts into five bins (pentiles), which captured the first fifth of the segments, the second fifth of the segments, and so on. We calculated semantic similarity scores between the written summaries and each pentile for each trial, using the three semantic similarity methods described above. This approach allowed us to investigate potential primacy effects, where earlier bins might show higher similarity, and recency effects, where later bins might show higher similarity.

**Fig. 9:**
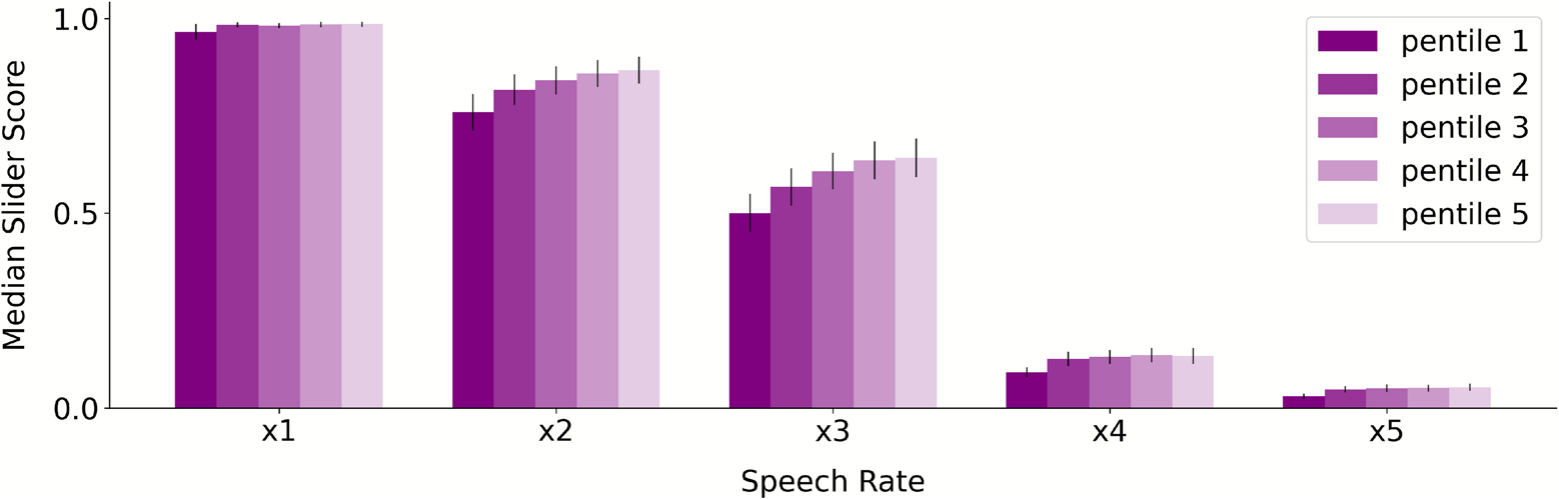
Slider scores over time per speech rate: To examine how comprehension changes over the course of trials, we divided the slider scores across trials for each time point into five bins (pentiles) and took their median for each speech rate. *Error bars* represent standard error

We conducted an analysis of variance (ANOVA) to examine the main effects of speech rate and bin, as well as their interaction, on semantic similarity scores. Results showed a significant main effect of speech rate (*F*(1,13746)= 2573.8, *p*<.001), but not of bin or an interaction, indicating that the GloVe-Written summary method does not detect an effect of information position, despite a numerical trend towards a recency effect (Fig. 8). We then explored whether the semantic similarity measures that predicted comprehension worse than GLoVe-Written Summary Segment capture memory effects: the GLoVe-Heard Segment and BERT semantic similarity scores. By contrast, following the same analysis approach, GLoVe-Heard Segment and BERT semantic similarity scores showed both the main effect of speech rate (GLoVe-Heard Segment: ${F}(1,7416)=2220.34$, *p*<.001;BERT: ${F}(1,7416)=595.15$, *p*<.001) and bin (GLoVe- Heard Segment: ${F}(1,7416)=6.81$, *p*<.01;BERT: ${F}(1,7416)=61.82$, *p*<.001). This suggests that the written summaries provided by listeners were indeed biased toward containing more information about the latter portions of the speech segment, but only certain methods of assessing semantic similarity are sensitive to detecting this effect.

While it is possible that the tendency for participants to include more information about the latter portions of heard segments could reflect a memory effect, it is also possible that participants actually *understand* the latter portions of the segments better, leading them to refer to them more in the written summaries. As static post hoc measures cannot distinguish between these two possibilities, we used our slider measure to test whether participants reported higher comprehension towards the end of the segment, indicating better understanding.

**Fig. 10:**
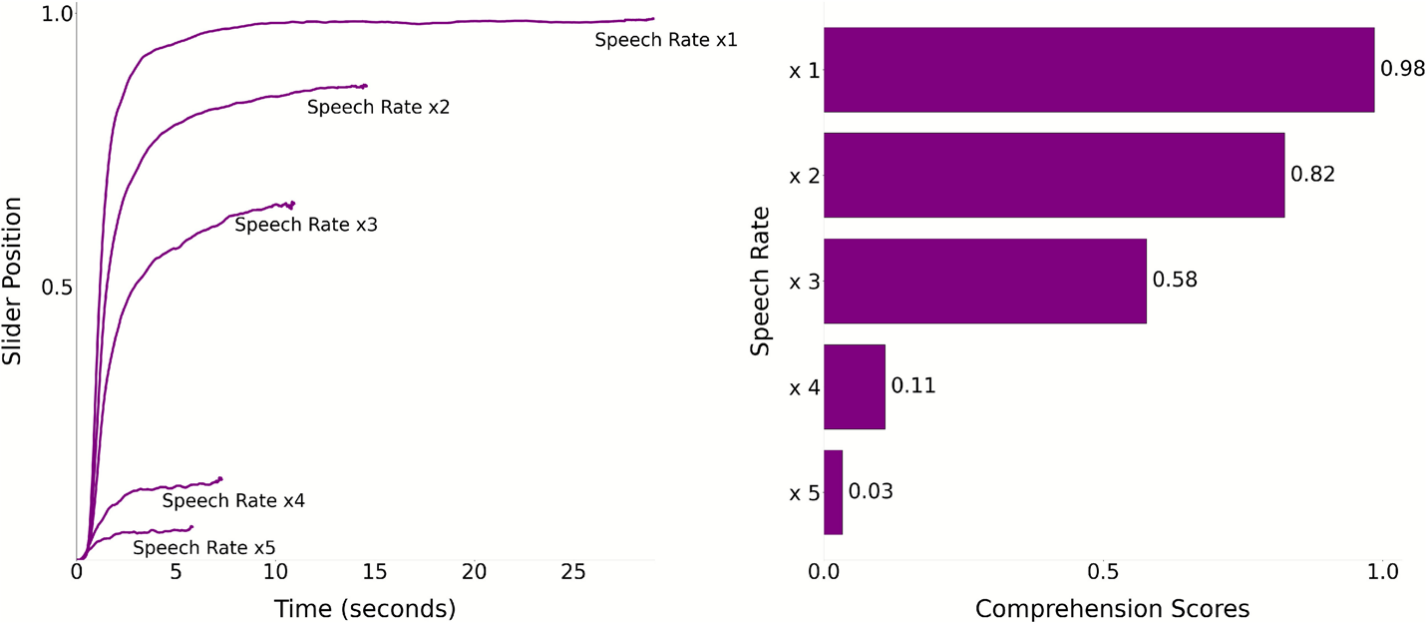
Slider scores per speech rate. *Left panel:* Slider scores reported via the slider on each time point are averaged across trials per speech rate. *Right panel:* Mean of the median slider scores across trials per speech rate

To do so, we first excluded the portion of the time course where participants were rapidly moving from the origin 0 starting point. Using the differential of average slider positions for each time point per speech rate condition (Fig. 10), we determined cut-off points for the initial movement: 5, 4, 2.5, 2, and 1.5 s for speech rates x1 to x5, respectively. We removed these initial segments and conducted further analysis on these cropped slider scores. We divided the slider scores into pentiles, similar to the analysis described above, such that we obtained an average slider score for the first fifth, second fifth, and so on. For each pentile, we calculated the median comprehension score to represent a single value per pentile. This allowed us to analyze comprehension trends over time for each speech rate condition.

To test whether comprehension scores significantly changed over the course of each trial, we conducted a two-way ANOVA with speech rate and pentiles as factors. The analysis revealed significant main effects for both speech rate (*F*(4, 525) =1003.88, *p* < .001) and pentiles (*F*(4, 525) = 4.28, *p* < .01). However, the interaction between speech rate and pentiles was not statistically significant (*F*(16, 525) = 0.60, *p* = .88). These findings suggest that comprehension changes as a function of the segment unfolding. Interpreting the ANOVA results in light of Fig. 9 demonstrates increased comprehension for the latter portions of audio segments. This pattern suggests that comprehension improved progressively throughout the segments, which could also explain participants’ increased likelihood of writing their summaries about the latter portions of the segments as compared to earlier portions of the segments. Further research should examine the relative contribution of real-time comprehension versus memory biases by employing paradigms that orthogonalize elapsed time in the segment from ease of comprehension.

#### Characterizing the comprehension decline

Finally, given the observed decline in comprehension with increasing speech rate across all measures, we post hoc decided to test whether this decline scales linearly with our experimental manipulation or follows a nonlinear pattern. A linear decline would suggest that speech comprehension primarily relies on sensory processing: As the sensory input speeds up, comprehension decreases linearly. In contrast, a categorical drop would support studies suggesting a bottleneck in the information-integration speed of the speech comprehension system (Vagharchakian et al., 2012), leading to successful comprehension up to a point, and then a dramatic decline.

To test whether slider scores varied linearly or categorically with changes in sensory input, we fit a linear model and sigmoidal models predicting median slider scores from speech rate. We fit models within a fivefold cross-validation. Folds were created over speech rates, ensuring that each fold has an equal number of trials consisting of each speech rate.

Linear model:

$$ y = \beta _0 + \beta _1 \cdot x $$

Sigmoidal model:

$$ y = \frac{\beta _0}{1 + \exp (-\beta _1 \cdot (x - \beta _2))} $$

Here, *y* represents the median slider scores predicted by the model, and *x* represents speech rate. In both models, $\beta 0$ serves as the intercept (analogous to the maximum asymptote in the sigmoid model), $\beta 1$ controls the slope (representing the growth rate in the sigmoid model), and $\beta 2$ denotes the center of the sigmoid curve, indicating where the main transition - drop in comprehension- occurs. The $\beta$ parameters were fit with Scipy’s curve_fit function (Jones et al., 2001).

**Fig. 11:**
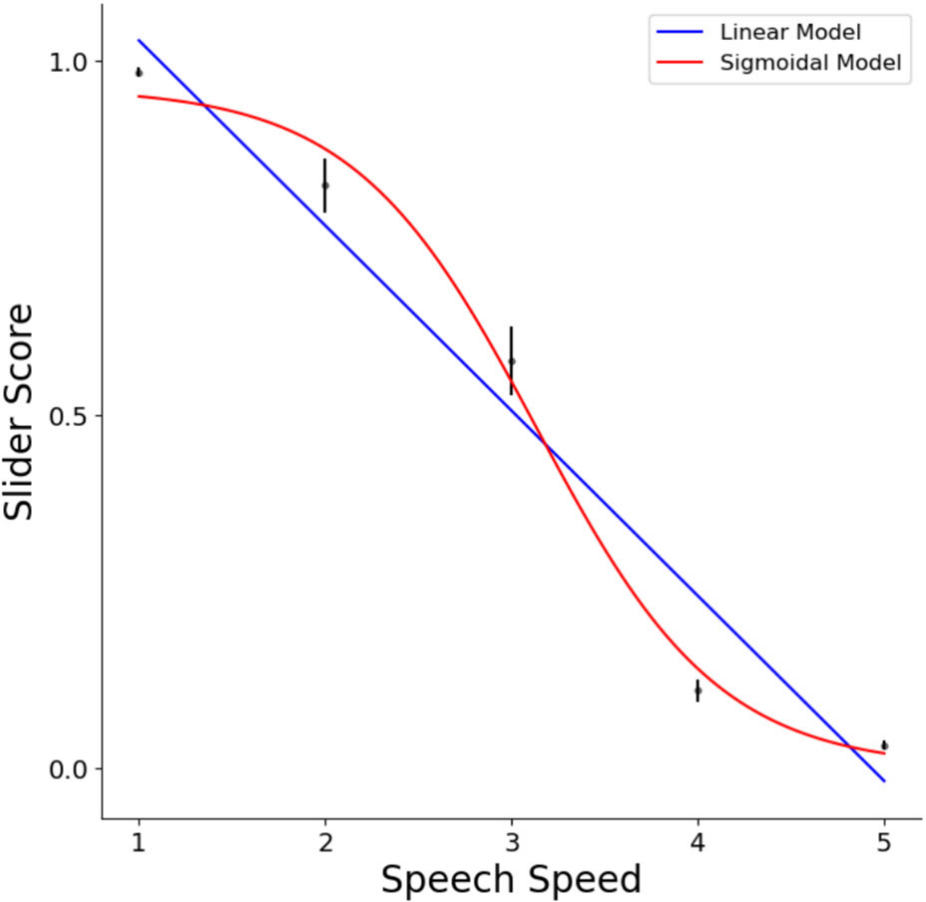
Linear vs. sigmoidal models. The *blue line* shows how the linear model predicts median slider scores of each trial, whereas the *red line* shows the sigmoidal model’s predictions. The mean of comprehension scores across trials for each speech rate is represented by *black dots*. *Error bars* show the standard error of the mean (SEM), calculated by averaging each participant’s response for each trial, then computing the overall standard error across these participant-trial averages

We calculated the Pearson correlation coefficients (*r*) between predicted and actual values for each model in each fold to assess prediction accuracy. The average correlation across folds suggested that the sigmoidal model (*mean MSE*= 0.043) outperformed the linear model (*mean MSE*= 0.039), showing a steeper decline after speech rate x3 (Fig. 10). Furthermore, a Wilcoxon test across subjects showed that both models’ predictions were above chance ($r > 0$) and that the sigmoidal model outperformed the linear model ($r_{\text {sigmoid}} > r_{\text {linear}}; p < 0.01$). This indicates that the sigmoidal model provided a better fit to the data than the linear model (Fig. 11). These results suggest that the decline in comprehension has a non-linear relationship to the change in speech rate.

## Experiment 2

In Experiment 1, we validated our novel measure by comparing it against commonly used post hoc comprehension measures and highlighted their limits: they are static, they cannot disentangle genuine comprehension from memory influences, and multiple-choice scores are vulnerable to variability in item difficulty and content familiarity. However, to accommodate these post hoc measures, we used short segments, leaving open the question of whether the slider generalizes to longer, continuous listening, typical of naturalistic experimental paradigms.

**Fig. 12:**
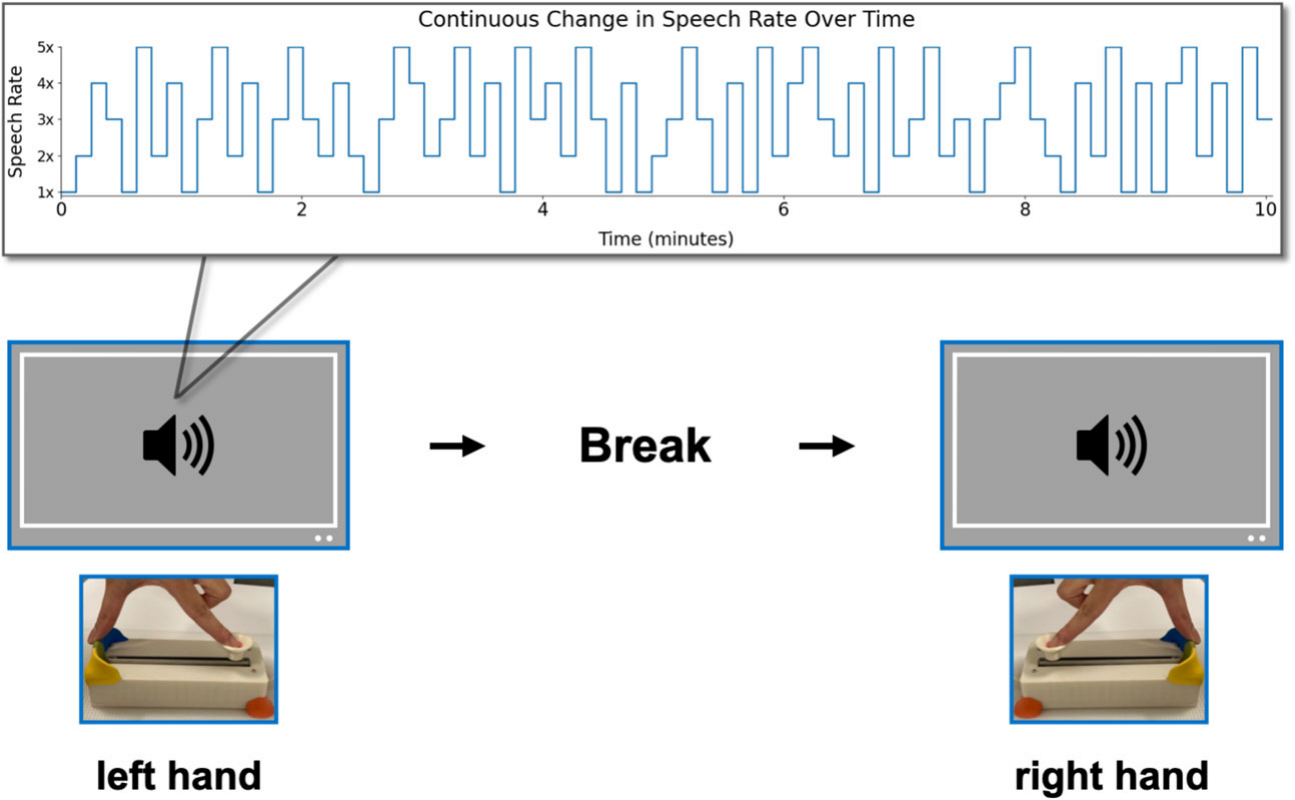
Experiment 2 paradigm. Participants listened to two continuous 10-s segments in which the speech rate varied, speeding up from $\times 1$ to $\times 5$

We addressed this question in Experiment 2 by testing the slider during extended, continuous listening. Participants used the slider to rate comprehension while listening to 10-min audio segments in which speech rate (x1-x5) varied every 7.5 s.

In addition, we tested practical considerations for future deployment with neuroimaging. A potential concern when using the slider in conjunction with neuroimaging methods is how to disentangle motor-related activity (from moving fingers to operate the slider) from stimulus-driven neural responses related to comprehension. This challenge is, of course, not unique to our device: button presses and similar motor responses are routinely collected during neural recordings as a way of collecting behavioral data during the neural recording session. A solution is to counterbalance responses across the left and right hands, which results in activity localized to the right versus left motor cortex, which critically is orthogonal to the experimental manipulation of interest. This lateralization allows motor-related activity to be distinguished from stimulus-driven signals (e.g., Grootswagers, Wardle, & Carlson, 2017; Gwilliams & King, 2020). To test whether behavioral responses provided with the left and right hands provide comparable measurements with the slider, in Experiment 2, we asked participants to switch hands midway through the session, allowing us to compare their responses.

### Methods

#### Participants

Thirty native English speakers without any diagnosed hearing or neurological disorders participated in Experiments 2 and 3 (21 females, *mean age*= 30.60 years, *age range*= 18–70 years). They received course credit or monetary compensation for their participation.

We implemented an attention check using the same criterion applied in Experiment 1: The distribution of slider movements across trials and participants must not exceed $\pm 3.5$ standard deviations from the mean. None of the participants were identified as outliers under this criterion, and therefore no data were excluded (see Supplementary Fig. 5). for individual participants’ slider reports).

**Fig. 13:**
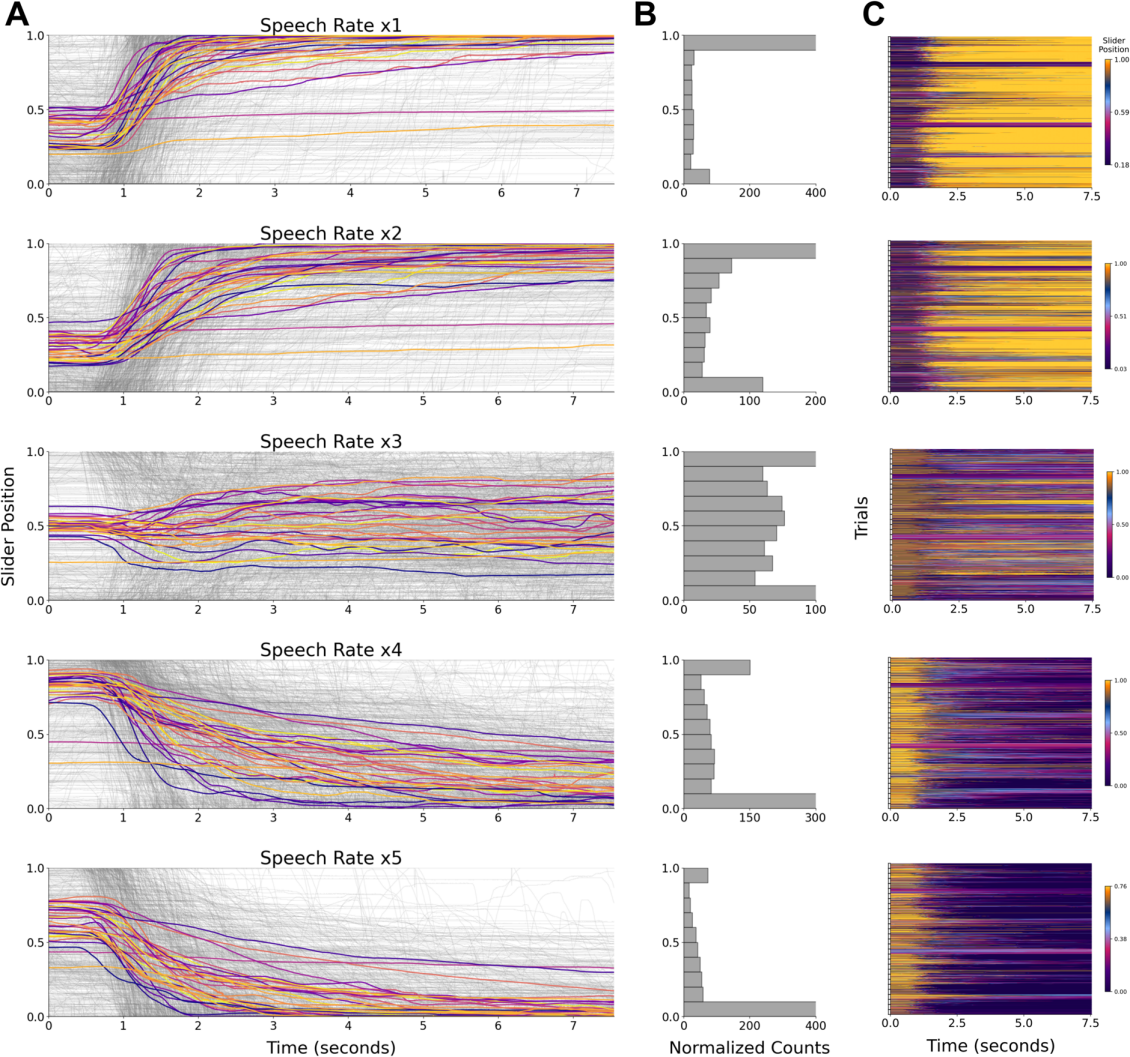
Slider responses by speech rate for Experiment 2. **A:** Normalized time-resolved slider scores for each speech rate. *Grey lines* represent individual trials, while *colored lines* show the average slider values across trials for each participant at each speech rate. The *x*-axis represents time in seconds, and the *y*-axis represents the values reported via the slider. **B:** The histograms depict how many times (*x*-axis) each value reported via the slider appeared across trials for each speech rate. The counts are normalized by dividing the number of values by the mean duration of the respective speech rate’s trials for visualization purposes. The values (*y*-axis) are grouped into ten bins. **C:** The slider position is plotted over each trial time course (*x*-axis). Each *bracket* marks the transition between participants, with each *row* representing a trial. The color scale’s minimum and maximum values are set to the 10th and 90th percentiles of slider positions for each speech rate condition

#### Materials

##### Stimuli

The same audiobook as Experiment 1, *Someday, Someday, Maybe* (Graham, 2014), served as the stimulus. We segmented the audio into 7.5-s clips and time–compressed them in Praat (Boersma & Weenink, 2024) to create five speech rate conditions: original speed and 2x, 3x, 4x, and 5x faster than the original recording rate. Importantly, segments were not shuffled: speeded clips were presented in their original narrative order so that each chunk followed logically from the preceding context. We maintained the constant pitch of the time compression while altering the speech rate. For sped-up conditions that had shorter segment durations, multiple compressed clips were concatenated to preserve the 7.5-s total duration (e.g., two adjacent 3.75-s segments for the x2 condition) so that we have equal duration of chunks for each speech rate. We presented the stimuli in chronological order of the story progression, but pseudo-randomized the speech rate conditions, with no direct repetition of consecutive conditions. Each condition appeared 16 times per block, with five conditions totaling 10 min per block [(16 $\times$ 5 conditions $\times$ 7.5s) $\div$ 60].

##### Design and procedure

Participants completed the control measures (Digit Span and Digit In Noise) before the main experiment. We presented two blocks, resulting in 32 presentations per condition across the experiment. Participants reported their comprehension continuously through the slider while listening to the 10-min audio segments, as they did in Experiment 1.

After the first block, we asked participants to switch the hand they were using to report comprehension through the slider. This was done so to ensure that the slider can be used with both the dominant and non-dominant hand, so that when employed with a neuroimaging device, motor artifacts and stimulus-driven artifacts can be differentiated by utilizing the laterality of motor responses (Fig. 12).

#### Analysis

We used Python version 3.11.8 (Van Rossum & Drake, 2009) for slider score visualizations, and RStudio version 2023.12.1+402 (Posit Team, 2024) for all other analyses and plots.

**Fig. 14:**
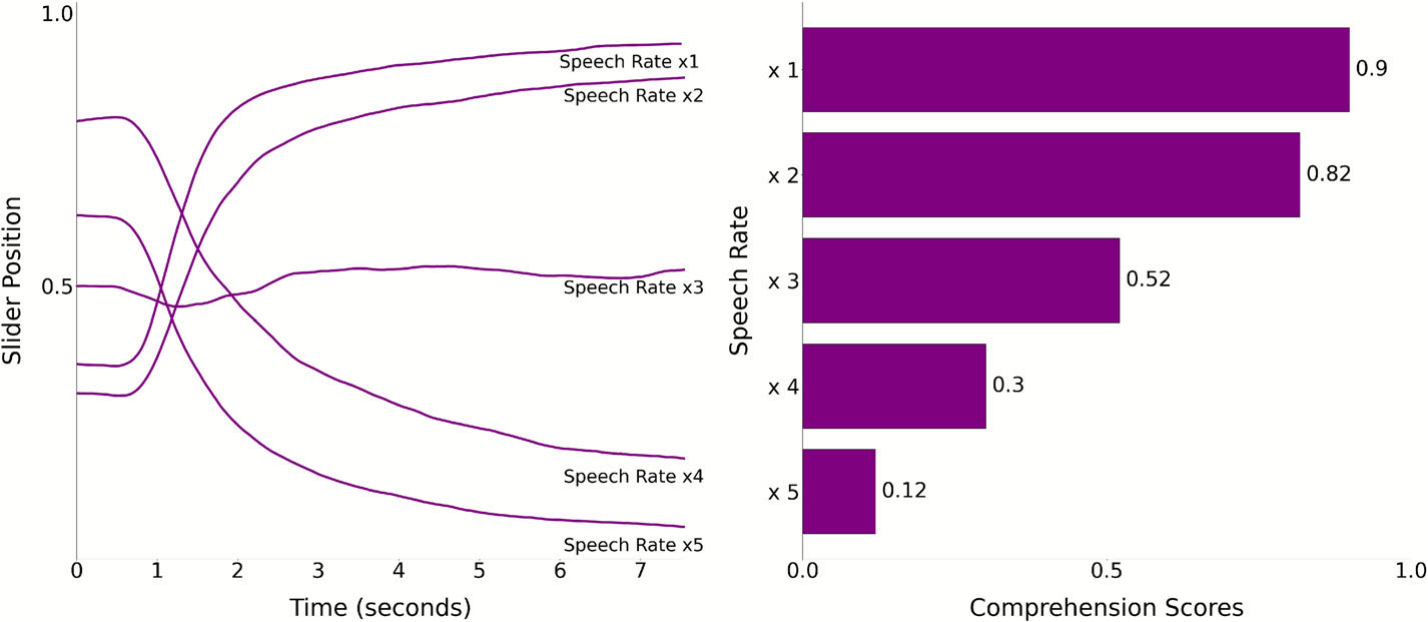
Slider scores per speech rate for Experiment 2. *Left panel:* Slider scores reported via the slider on each time point are averaged across trials per speech rate. *Right panel:* Mean of the median slider scores per speech rate

### Results

#### Slider scores

To test whether median slider responses differ across speech rates, we fit a linear mixed-effects model predicting median slider scores from speech rate, with random intercepts for working memory and digit in noise. Replicating Experiment 1, faster rates were associated with progressively lower responses: x2: $\beta = - 0.082$, ${SE} = 0.012$, ${t}(4780.84) = -6.54$, *p* < .001; x3: $\beta = -0.378$, ${SE} = 0.012$, ${t}(4780.84) = -30.30$, *p* < .001; x4: $\beta = -0.598$, ${SE} = 0.012$, ${t}(4780.84) =-47.96$, *p* < .001; x5: $\beta = -0.779$, ${SE} = 0.012$, ${t}(4780.84) = -62.50$, *p* < .001 Fig. 13. Tukey-adjusted pairwise comparisons among all five speech-rate levels were significant (all *p* < .0001), indicating that slider scores at each speech rate differed from every other rate (Fig. 14).

#### Investigating effects of the used hand on operating the slider

To assess whether median slider scores depend on speech rate or the hand used to operate the slider, we fit a linear mixed-effects model with fixed effects of speech rate and hand used to engage with the slider, and random intercepts for working memory and digit in noise:

$$\begin{aligned} \texttt{lmer}&\Big (\mathtt {median\_slider\_score} \sim \mathtt {speech\_rate}\\&* \mathtt {hand\_used} + (1 \mid \mathtt {digit\_span\_score}) \\&+ (1 \mid \mathtt {digit\_in\_noise\_score})\Big ) \end{aligned}$$

An ANOVA over the model revealed a main effect of speech rate, *F*(4, 4775.8) = 145.53, *p* < .001, but no main effect of hand, *F*(1, 4775.8) = 0.44, *p* = .51, and no interaction, *F*(4, 4775.8) = 0.72, *p* = .58. These results demonstrate that motor related activities introduced by slider use can be dissociated from comprehension-related neural signals by alternating the laterality of motor actions. This supports the feasibility of co-registering the device with neuroimaging methods (Supplementary Fig. 6).

**Fig. 15:**
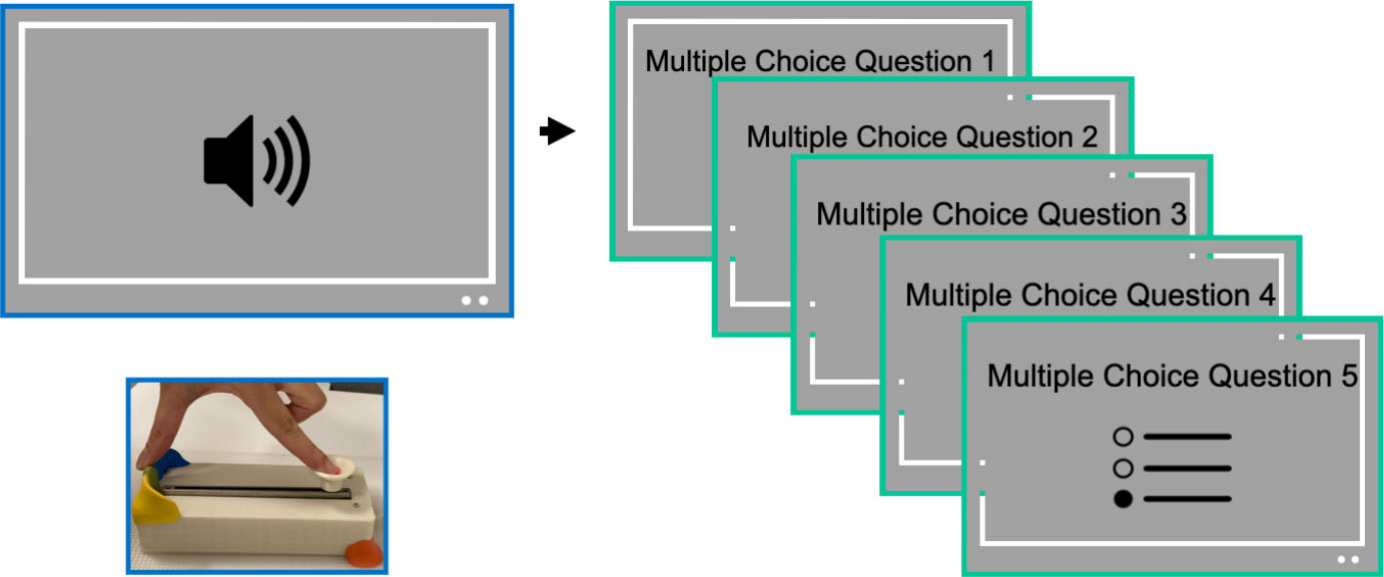
Experiment 3 paradigm. Participants heard four stories twice (x1 and x2.5) in a pseudo-randomized order without immediate repeats; two began at x1 and two at x2.5. On each trial, they were cued to use or withhold the slider, yielding four slider trials (two per speed) and four no-slider trials. After each segment, they answered five multiple-choice questions

## Experiment 3

In Experiments 1 and 2, we established that the slider provides a dynamic behavioral readout of speech comprehension, operates robustly in naturalistic, continuous listening settings, and is suitable to use simultaneously with neural recordings. However, in both experiments, we targeted a single comprehension manipulation (speech rate), leaving open whether the measure can track fluctuations in comprehension that are due to factors other than speech rate. Therefore, in Experiment 3, we tested whether our measure can track multiple challenges by manipulating both speech rate and information load within stories (high- vs. low-surprisal moments).

Furthermore, we aimed to test whether using the slider while listening to speech may actually interfere with the comprehension process itself. To assess this, we compared multiple choice question accuracy when using the slider and when not.

Finally, in Experiments 1 and 2, we summarized the slider scores over time, in order to make them comparable to the post hoc measures of interest. Here, we additionally apply a time-resolved analysis approach – temporal response function (TRF) modeling – to examine the full dynamic time course. This also allowed us to estimate participant-specific response delays, to demonstrate that these can be characterized and incorporated into future neuroimaging analyses.

### Methods

#### Participants

The same participants who completed Experiment 2 also participated in Experiment 3 in the same session. We eliminated four participants’ data because they failed the multiple-choice question accuracy criterion we preregistered for Experiment 1.

#### Materials

##### Stimuli

We selected four children’s fables (*The Fox and the Grapes*, *The Ant and the Grasshopper*, *The Wind and the Sun*, and *The Cat and the Mice*) from (Aesop, 2023) as source material. We adjusted each story so that the passage alternated between lower-surprisal parts and higher surprisal parts. Higher surprisal is associated with higher information load, and is considered harder to understand (Gwilliams & Davis, 2022). Each part was about 30 words long, but the story was recorded as a single continuous story without breaks.

We estimated surprisal using a GPT-2 language model (Vaswani, 2017). We confirmed that the surprisal values in the low- and high-surprisal parts differed significantly across stories using a paired-samples *t*-test (*t(3)*=7.56, *p*<.01, Cohen’s *d*=3.78, $M_{\text {low}}=4.91$, $M_{\text {high}}=5.90$).

A single English native speaker recorded all stories. To create the fast condition, recordings were time-compressed to 2.5x using Praat’s changevtpitchduration.praat (mapping new_dur and original_dur to segment duration $\times \,1/$speeding factor) while preserving pitch (Boersma & Weenink, 2024).

To further promote variability in comprehension success, the eight audio files (four stories $\times$ two speeds) were mixed with speech-weighted noise. The speech-to-noise ratio was +3.5 dB, where speech RMS level exceeded the speech-weighted noise RMS level by a factor of 1.5. The original (x1) durations were: Fox 148.25 s, Wind 140.66 s, Grasshopper 181.76 s, Mouse 155.41 s; the corresponding x2.5 durations were: Fox 60.59 s, Wind 56.31 s, Grasshopper 72.67 s, Mouse 62.36 s. In total, the stimulus set comprised eight audio tracks (four stories at x1 and the same four at x2.5).

##### Design and procedure

In Experiment 1, we presented each segment once to avoid performance gains from repetition rather than genuine changes in comprehension. A potential drawback is item heterogeneity, whereby some segments may simply be harder than others. However, because the same segments were presented in different speech rates across participants, the aggregate analyses are unlikely to be affected by item heterogeneity. In Experiment 3, we addressed this directly by presenting the same segments at both x1 and x2.5 to each participant (an analysis on order effects can be found in Supplementary Materials).

Story order and speed order were pseudo-randomized within participants. The same story was not repeated directly after each other. We ensured that of the four stories, two were initially presented at x1 speed, and the other two were presented in the opposite order. Before each trial, participants were instructed whether or not to use the physical slider during the upcoming audio. Across the session, each participant used the slider on four trials (two x1 and two x2.5) and withheld slider responses on the remaining four trials. This allowed us to evaluate whether using the slider interfered with comprehension.

After each audio, participants answered five multiple-choice questions about the story they had heard. For each story, a pool of ten questions was available: the first presentation of a story sampled five questions at random, and the second presentation used the remaining five, ensuring no question was repeated within participants. The experiment was implemented in PsychoPy (Peirce et al., 2019) (Fig. 15).

**Fig. 16:**
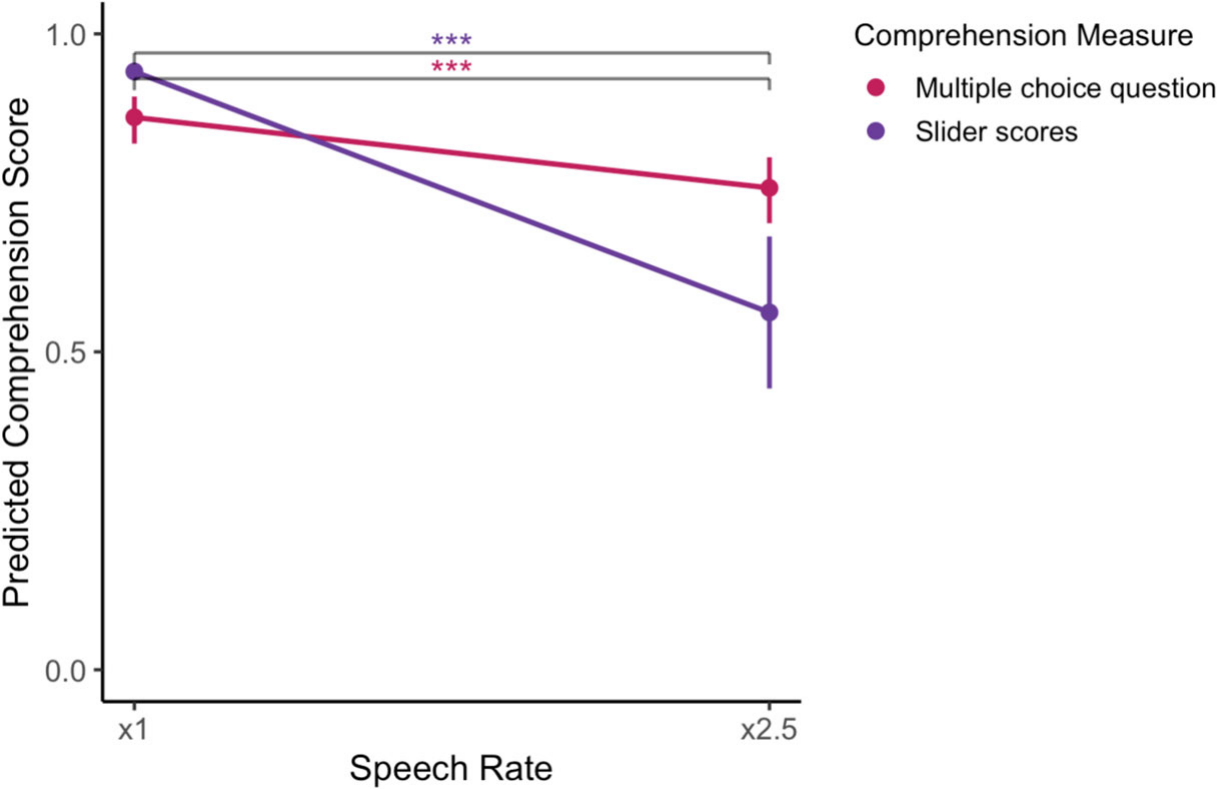
Comprehension declines at the faster speech rate across measures. *Points* and *lines* show estimated marginal means from a binomial GLMM (multiple-choice accuracy, *in pink*) and a linear mixed-effects model (slider scores, *in purple*). *Error bars* are 95% confidence intervals. *Brackets* indicate the x1 vs. x2.5 contrasts; both measures are significantly lower at x2.5 (*** = p<.001). *Axes* display speech rate (*x*-axis) and predicted comprehension score (*y*-axis)

#### Analysis

We used Python version 3.11.8 (Van Rossum & Drake, 2009) for temporal response function (TRF) analysis and plots, and RStudio version 2023.12.1+402 (Posit Team, 2024) for all other analyses and plots.

### Results

We took the median time course of slider scores to have a single comprehension score for each trial. In each regression model, we included the digit span score and digit-in-noise scores as random slopes to account for within-subject variability in comprehension scores that is not due to comprehension per se. We used the lme4 R package (Bates et al., 2015) for all regression analysis.

#### The change in comprehension scores with increased speech rate

We tested whether slider scores and multiple-choice accuracy decrease with increasing speech rate, as observed in prior experiments. We first fit a binomial generalized linear mixed-effects model predicting multiple-choice accuracy from speech rate (x1 vs. x2.5). Multiple choice question accuracy was significantly lower at x2.5 than at x1 (for speech rate= x2.5 ($\beta = -0.75$, ${SE} = 0.16$, ${z} = -4.59$, *p*<.001). We then fit a Linear Mixed-Effects Model to test the same effect on slider scores. As we expected, there was a significant main effect of speech rate, indicating that the median slider scores differed between the x1 and x2.5 conditions (${F(1,507.41)}=497.80$, *p*<.001). The x2.5 condition yielded a significantly lower median slider score compared to the x1 condition ($M_{\text {diff}} = -0.38$, ${SE} = 0.017$, ${t}(509)=-22.31$, *p*<.001). These results replicate the speech-rate effect observed in Experiments 1 and 2: faster speech reduces comprehension (Fig. 16).

#### Investigating whether using the slider alters comprehension

Next, to test the possibility that the act of using the slider interferes with comprehension, we compared multiple-choice accuracy between trials where participants used the slider and where they did not. We fit a binomial generalized linear mixed-effects model predicting multiple-choice question accuracy from a fixed effect for slider use (present vs. absent). Slider use did not affect accuracy (present: $\beta = -0.21$, ${SE} = 0.16$, ${z} = -1.34$, ${p} = .18$). Thus, we find no evidence that operating the slider while listening changes comprehension.

**Fig. 17:**
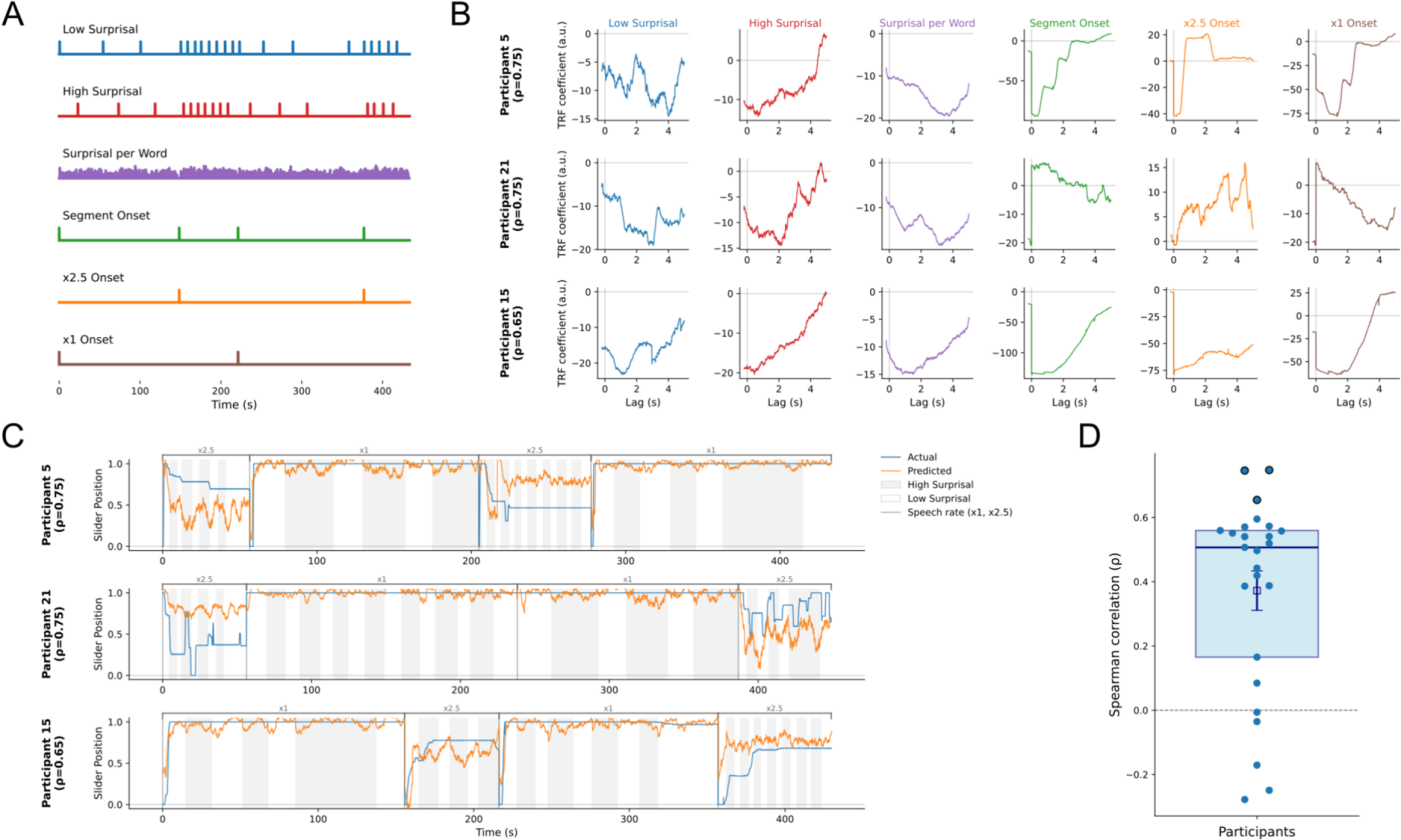
TRF analysis **A.** Pulses of each feature in the TRF analysis. **B.** By-participant variation in response delays for three participants. *Rows* correspond to participants and columns to annotation features: (1) low surprisal chunk onsets, (2) high surprisal chunk onsets, (3) surprisal per word (each word’s surprisal value), (4) segment onset, (5) onset of x1 speech rate segment, and (6) onset of x2.5 speech rate segment. The *y*-axis shows the TRF coefficients (*y*-axis) estimated over lags from -0.2 to 5 s (*x*-axis). You can find ground truth slider positions and predicted positions for all participants in Supplementary Materials (Supplementary Figs. 8 & 9, respectively.) **C.** Actual vs. predicted slider responses for three participants. Each panel shows the actual slider position (blue) and the position predicted by the TRF model (*orange*) across the four stories in which the participants operated the slider while listening. *Brackets* above the traces indicate the speech rate for each story (x1 or x2.5). *Shading* within stories marks surprisal: *white bands* denote low-surprisal segments and *gray bands* denote high-surprisal segments. **D.** Box scatter plot of per participant Spearman correlations ($\rho$) between the slider responses predicted by the TRF model and the observed slider traces. The *points* show individual participants; the three participants shown in B and C are marked with *black circles*. The *box* indicates the interquartile range, the *whiskers* the standard error of the mean, the square the mean, and the *dark blue line* the median

#### Modeling individual response latencies & tracking multiple comprehension manipulations

Finally, to leverage the time-resolved nature of our measure, we modeled participants’ slider scores as a function of our comprehension manipulations using a temporal response function (TRF) analysis. TRF analysis is widely used in cognitive neuroscience research to characterize how continuous neural signals track time-resolved naturalistic stimuli such as speech and music (for example, see Di Liberto et al., 2020; Gwilliams, Marantz, Poeppel, & King, 2025; Kries et al., 2024). Here, we took the novel approach of applying the TRF analysis to the behavioral time series - participants’ continuous slider responses - to estimate how multiple features of the audio segments drive moment-by-moment comprehension. To the authors’ knowledge, this is the first use of TRF models to analyze and interpret time-resolved behavioral language processing data.

The analysis pipeline comprised four steps to estimate TRFs between the audio segments and slider responses. First, we obtained precise word-level timestamps using CharSiu forced alignment (Zhu, Zhang, & Jurgens, 2022). Second, we constructed six stimulus annotation vectors, as explained below. Third, we concatenated the slider responses and annotation vectors across stories, in the order that they were presented to the participant. Fourth, we performed TRF analysis using a kernel window of -0.2 to +5 s using MNE’s ReceptiveField with ridge regularization ($\alpha =1.0$) and 3-fold cross-validation. For participants who did not have enough variation within a fold, we conducted a 2-fold cross-validation instead. We fit a time-lagged ridge regression from the annotation vectors to the slider responses, predicted held-out responses, and quantified accuracy as Spearman’s $\rho$ between predicted and observed signals.

The six stimulus annotation vectors were created separately for each of the eight stimuli (Fig. 17A). They were created as follows: For each of the six annotations, a vector of zeros of the length of the slider data was initiated, and then the impulses of each feature were placed at the right time point:

1. *Low surprisal condition onset*: a value of 1 was attributed to the beginning of each low-surprisal passage, which corresponded to the beginning of the first word in that passage. The first word’s onset timestamp was extracted using the forced aligner (CharSiu).
2. *High surprisal condition onset*: a value of 1 was attributed to the beginning of each high-surprisal passage, which corresponded to the beginning of the first word in that passage. The first word’s onset timestamp was extracted in the same way as for the low-surprisal condition onset vector.
3. *Word-by-word surprisal*: GPT2-derived surprisal values were attributed at the positions of their respective word’s onset. Surprisal values were estimated using a GPT-2 language model (Vaswani, 2017).
4. *Segment onset*: a value of 1 was attributed to the first position, whereas all the other values remained 0s.
5. *x1 speech rate condition onset*: a value of 1 was attributed to the first position whenever the story corresponded to x1 speech rate.
6. *x2.5 speech rate condition onset*: a value of 1 was attributed to the first position whenever the story corresponded to x2.5 speech rate.

For group-level inference, we tested whether per-participant $\rho$ exceeded zero (chance) with a one-sample *t*-test: ${M}=0.38$, ${SD}=0.33$, yielding ${t(24)}=5.80$, *p*<0.001 (Fig. 17D). These results indicate that the six stimulus features robustly predict real-time comprehension across participants (Fig. 17B). We also show that the TRF model can capture the individual differences in slider response delays by visualizing the learned kernel for individual participants (see Supplementary Fig. 17C).

Next, to test whether modulations in surprisal lead to measurable differences in slider scores, we re-fit the TRF models after removing the three surprisal-related features (low-surprisal onsets, high-surprisal onsets, and word-by-word surprisal) and compared the two model performances (with vs. without surprisal). We conducted a one-sample *t* test on the per-participant differences (with minus without surprisal) on the $\rho$ values to assess whether the mean improvement differed from zero. Surprisal features significantly improved model performance: mean $\rho _{\text {with surpisal}} = .37=$, mean $\rho _{\text {without surprisal}} = .20$, mean $\Delta \rho = .18$; one-sample ${t}(24) = 2.71$, *p* < .01, Cohen’s $d = 0.54$. These results indicate that the slider captures context manipulations such as word surprisal in a time-resolved manner.

## Discussion

The cognitive and neural processes upholding speech comprehension are an important and active area of research, with translational consequences for neurological disorders that impact language function. A major challenge for this line of work is simply put: How do you measure comprehension? Without a reliable behavioral method of determining how much a person understands, including fluctuations in understanding moment by moment, it is difficult to anchor cognitive and neuroscientific processes with the outcome of interest - successful understanding.

Aiming to close that gap, we designed and tested a novel way of measuring speech comprehension in real time that is compatible with neuroimaging methodologies. Across three experiments, we validated slider performance against the post hoc static measures that have been commonly used in the field to date, and found that it was able to better capture fluctuations in comprehension due to speech rate and information load. Furthermore, we tested that the slider can be counter-balanced across right and left hands to reduce motoric confounds, and that engaging with the slider does not interfere with the comprehension process itself. Finally, we show that time-series modeling approaches, such as temporal response function (TRF) models, can be used to capture comprehension behavior in real time during continuous, uninterrupted speech.

One of the challenges of validating a measure of comprehension is that there lacks a ground truth - there is no way of knowing how much someone truly understands, against which to compare a measure of understanding. In lieu of a ground truth, we employed speech manipulations that have been shown to modulate *relative* comprehension success; namely, speech rate (Lubinus et al., 2023; Nourski et al., 2009; Vagharchakian et al., 2012) and surprisal (Gwilliams & Davis, 2022). There are other methods that could have been chosen here, too, such as varying the signal-to-noise ratio. Across the three experiments, we find that both speech rate and surprisal lead to measurable fluctuations in slider reports. This supports that the slider device can be used to measure comprehension challenges arising from a range of sources.

Another approach we took to validate the slider as a method of measuring comprehension was to compare slider responses to a range of post hoc measures that are commonly used in the field. In doing so, we also accounted for participants’ working memory capacity and auditory acuity to ensure that comprehension was assessed beyond individual variations (Billings et al., 2023; Emmorey et al., 2017; Lubinus et al., 2023; Taylor, 2003; Tun et al., 1991). We found that all comprehension measures significantly predicted speech rate, with comprehension scores decreasing as speech rate increased. Notably, our novel measure was the predictor with the highest coefficient among all the measures, capturing the decline in comprehension more effectively than traditional post hoc assessments (Fig. 7).

One potential concern is that using the slider while listening could interfere with or distract participants from fully comprehending. To test this, in Experiment 2, we compared multiple-choice question accuracy for segments completed with versus without the slider. Importantly, accuracy did not differ between conditions, suggesting that using the slider does not interfere with comprehension.

In addition to validating our real-time measure, we confirmed a number of limitations of using post hoc comprehension measures. Multiple choice question response accuracy was above the chance level of 25%, even at the highest speech rate (Fig. 6f). Yet, participants consistently reported that they did not understand those fast segments, based on ten-point scale ratings, and low similarity in their written summaries to the segments. One possible explanation is that participants might become familiar with the story, as all segments were from the same audiobook, albeit presented in a random order. They might be accumulating knowledge and predicting the correct answers based on previous segments or relying on their general world knowledge, allowing them to answer correctly without necessarily understanding the segments (McKenna, 2019; Wong, Denny, Luxton-Reilly, & Whalley, 202). Thus, a participant may be able to respond to multiple-choice questions above chance, even though they do not actually understand the segment that the question refers to.

There are further limitations to the multiple-choice question design. For instance, the level of accuracy obtained can vary depending on the nature of the questions asked and how they are presented (Al-Faris, Alorainy, Abdel-Hameed, & Al-Rukban, 2010; Ibbett and Wheldon, 2016). For continuous stimuli composed of multiple sentences, creating questions that represent the entire segment while maintaining consistent difficulty across questions is challenging. Employing more than one question per segment could reduce sample bias, but would result in very long experiments and still would not ensure consistency of challenge across questions.

To examine potential memory constraints reflected in participant summaries, we tested whether there are any primacy and recency effects (Richardson, 2007). There was a non-significant trend for the recency effect for GloVe- Written Summary semantic similarity scores. We chose to use semantic similarity scores calculated using this method in our analysis because they explained the most variance in comprehension. Interestingly, the recency effect was significant for the semantic similarity scores calculated using the other two methods (Fig. 8). One possibility is that GloVe- Written Summary semantic similarity scores are comparatively less sensitive to recency-driven memory biases, which may explain their stronger alignment with the ten-point scale measure. Alternatively, it is also possible that the other two methods capture genuine memory biases in participant summaries, which could account for their lower performance, which remains an open question for future work.

This trend highlights a limitation of measuring comprehension via summaries, as they reflect not only how much participants understood but also how much they could recall. Specifically, the final content of the audio segments was more frequently represented in participants’ summaries. This pattern could arise for two reasons, which post hoc measures cannot distinguish: (1) summaries might be biased toward the final content due to recency effects, with different encoding methods amplifying this bias to varying degrees, or (2) participants may genuinely understand the latter parts of the audio segments better.

To disambiguate these explanations, we analyzed the trajectory of slider scores for each speech rate using a similar pentile binning approach. We observed significant effects of both speech rate and pentiles on median slider scores (Fig. 9). These findings suggest that participants’ comprehension genuinely improved over the time course of the segments. Importantly, post hoc measures like summaries cannot differentiate between true comprehension and memory-related artifacts, whereas our novel real-time comprehension measure provides this critical distinction.

Our post hoc comprehension measures results suggest that the methods traditionally used to evaluate comprehension suffer from critical shortcomings. Besides being static and unable to capture dynamic changes in comprehension during natural listening, limitations include the inability to distinguish between genuine comprehension and memory influences (Fig. 8), and the susceptibility of multiple-choice questions to variability in question difficulty and participants’ familiarity with the content (Supplementary Fig. 1). Overall, our work makes the limitations of post hoc assessment explicit and provides the first method for recording speech comprehension success in real time.

Following the observation that comprehension declines with increasing speech rate across all measures, we analyzed the nature of this decline and found that it is better explained by a non-linear model rather than a linear one (Fig. 11). This categorical decline we observed supports previous findings showing that speech processing involves more than just the sensory system (Broderick, Anderson, & Lalor, 2019; Gillis et al., 2023; Gwilliams, Linzen, Poeppel, & Marantz, 2018; Hickok & Poeppel, 2007), as the linear decline in sensory input cannot explain the decrease in comprehension alone. This is likely due to a threshold of speech comprehension beyond which binding information to form comprehensive linguistic representations is not possible (Hagoort, 2005; Vagharchakian et al., 2012). Our findings complement previous fMRI research on sentence comprehension, suggesting that there are both linear and nonlinear responses to accelerated speech across different brain areas. Vagharchakian et al. (2012) demonstrated that in modality-specific sensory areas, activation varied linearly with stimulus duration. This is not surprising, considering that the input is a linearly accelerating signal. What was interesting is that in a large modality-independent left-hemispheric language network, including the inferior frontal gyrus and the superior temporal sulcus, they observed a time-invariant response followed by a sudden collapse for unintelligible speech stimuli. These findings suggest that in spoken sentence comprehension, higher stages of language processing operate at a fixed speed, imposing a temporal bottleneck after which binding incoming information is not possible. Our behavioral findings in spoken narrative comprehension support these results, while the novel method we propose here enables further questions to be answered.

An important component of a real-time measure of comprehension is that reports should fluctuate moment by moment as understanding itself wanes. As evidenced by the distribution of the slider movements across trials in Experiments 1 and 2, and by the fluctuations observed in individual trials, we find that participants engage with the slider dynamically as they listened to audio segments (Figs. 5 & 13). Furthermore, when applying TRF models to the slider responses in Experiment 3, we are able to capture dynamic changes in slider positions as a function of ongoing information in the stimulus. Because the TRF is fit on each participant’s data separately, this also allowed us to account for temporal differences across participants (Supplementary Fig. 9).

In future studies, using high-resolution EEG or MEG, we can investigate which processing bottleneck impedes comprehension. This can allow us to see when, how, and at which level of linguistic representation the deterioration occurs (Davis & Johnsrude, 2003; Gwilliams, Marantz, Poeppel, & King, 2023b), with a causal link to behavioral reports of comprehension. In other words, we can investigate the point where speech comprehension fails, and determine which levels of linguistic representation start to overlap and result in this failure (Gwilliams et al., 2025).

When using the slider with neural recording techniques, separating stimulus-driven responses from motor activity is important. Common methods of providing behavioral responses, such as pressing a button, or moving our slider, elicit neural activity associated with the hand and finger movements. A common practice in human neuroimaging to address this issue is to counterbalance responses across hands. This results in activity localized to the left versus right motor cortex, allowing researchers to differentiate finger-movement-related activity from stimulus-driven activity. In other words, laterality of motor activity is orthogonalized from the experimental features of interest (see, for example, Grootswagers et al., 2017; Gwilliams & King, 2020). To demonstrate that the same principle could be applied to our device, in Experiment 2 we asked participants to switch hands after the first half of the experiment. The responses collected with the dominant and non-dominant hand did not differ significantly, suggesting that motor activities can be accounted for utilizing the laterality of motor activity (Supplementary Fig. 6).

In addition to the limitations of post hoc comprehension assessments discussed above, the lack of appropriate tools to analyze natural listening comprehension data has traditionally led researchers to study language comprehension using isolated words (Bonilha et al., 2017; Gwilliams et al., 2018) or sentences (Davis et al., 2005; Lubinus et al., 2023). However, these approaches do not accurately represent how language is processed in everyday life and likely fail to capture the full richness of meaning derived from natural language. While there are studies on speech comprehension using continuous narratives (Brodbeck, Hong, & Simon, 2018; Chalas et al., 2024; Gwilliams et al., 2025; Zioga, Weissbart, Lewis, Haegens, & Martin, 2023), manipulating and measuring comprehension in a time-resolved manner has been challenging due to the aforementioned limitations.

The novel real-time comprehension measure proposed here addresses these challenges and enables the investigation of the causal neural mechanisms of naturalistic speech comprehension when combined with neuroimaging methods. Traditionally, neuroimaging research on speech comprehension has compared intelligible speech against non-linguistic, language-like auditory inputs (e.g., jabberwocky or backward speech) or languages that participants do not understand (see Fedorenko, Ivanova, & Regev, 224; Gillis et al., 2023 for examples). This approach was used to localize language-related brain regions or to differentiate neural activity corresponding to comprehension from non-comprehension, as there was no way to measure the level of comprehension during neural activity measurement. However, people can fail to understand speech for various reasons, such as pace or information density (see Mattys et al., 2023 for a review), even in a language they know.

To investigate whether the slider can be used to track different sources of comprehension difficulty, in Experiment 3, we manipulated word surprisal within audio segments as well as the speech rate. We showed that the slider can successfully track these different comprehension manipulations: the stimulus features, including speech rate and surprisal, robustly predict time-resolved slider scores across participants, and the modulations in surprisal lead to measurable differences in slider scores (Fig. 17). In future research, by combining time-resolved brain activity measures in response to speech (a dynamic signal) with time-resolved behavioral reports of comprehension, we can compare neural activity associated with successful speech comprehension under various challenging conditions. Ultimately, we can gain insight into the causal neural mechanisms underlying speech comprehension by observing which language representations are generated only during successful comprehension and their dynamics in a temporally resolved manner.

Overall, our findings demonstrate the validity of our novel time-resolved comprehension measure. We showed that it is possible to derive an online behavioral measure of speech comprehension in real time, a lack that has been a major limitation of naturalistic language studies. This overcomes numerous limitations of static post hoc assessments, including the inability to disentangle recency bias from comprehension for summarization, and challenges of multiple-choice question design. We propose that this continuous speech comprehension measure can be effectively integrated with neuroimaging techniques, offering a valuable tool for future research on dynamic processes during naturalistic listening, as well as other research endeavors looking at neural correlates of dynamic processes.

## Supplementary Information

Below is the link to the electronic supplementary material.

Supplementary file 1 (pdf 19302 KB)

## Data Availability

Data is available at https://osf.io/zk3c5/files/osfstorage, and code and experiment files are publicly available at https://github.com/irmak-ergin/measuring_naturalistic_speech_comprehension_2024. You can find the preregistration and link to the GitHub repository on doi.org/10.17605/OSF.IO/F5UZ8 as well. The design, analyses, and participant-exclusion criteria for Experiment 1 were preregistered. The only exploratory analyses not preregistered were the recency analysis and the investigation of the nature of decline in comprehension (linear vs categorical decline). Experiments 2 and 3 were conducted after preregistration, during the review process.

## Declarations

### Declaration of AI-assisted technologies

As mentioned in the “Methods” section, we initially explored the use of generative AI for preparing multiple-choice questions but ultimately abandoned this approach. During the preparation of this manuscript, AI-assisted technologies were used only for minor editing purposes (e.g., suggestions for grammar correction). All content was prepared and reviewed by the authors.
