# Supplementary material for: Measuring naturalistic speech comprehension in real time
Source: Behav Res Methods. 2026 Mar 27;58(4):93. doi: 10.3758/s13428-026-02941-1 (PMC13031255; doi:10.3758/s13428-026-02941-1)
Supplement: Supplementary file 1 — (pdf 19302 KB) [file 13428_2026_2941_MOESM1_ESM.pdf]

Supplementary Materials

1 Experiment 1

1.1 Excluded Participants

1.1.1 Exclusion Criteria

Before running analyses to answer our research questions, we wanted to make sure that our participants were engaged in the task. To do so, we a priori decided on a number of exclusion criteria and preregistered them. First, we excluded participants who failed to achieve a minimum comprehension threshold of 75% correctness on multiple-choice questions at the slowest speech rate (Supplementary Fig. 1). We eliminated 8 participants' data because they failed this criterion. The vast majority of questions were answered at ceiling among participants who passed this criterion, with a subset proving more difficult, as reflected by accuracy in the non-sped-up (speech rate x1) condition (Supplementary Fig.2). Our other exclusion criteria were: no significant differences in ratings on a 10-point scale between the slowest and fastest speech rates; failure to provide a summary across experimental conditions; distribution of the amount of slider movements across trials and participants exceeds ± 3.5 standard deviations from the mean. No participants failed these other criteria.

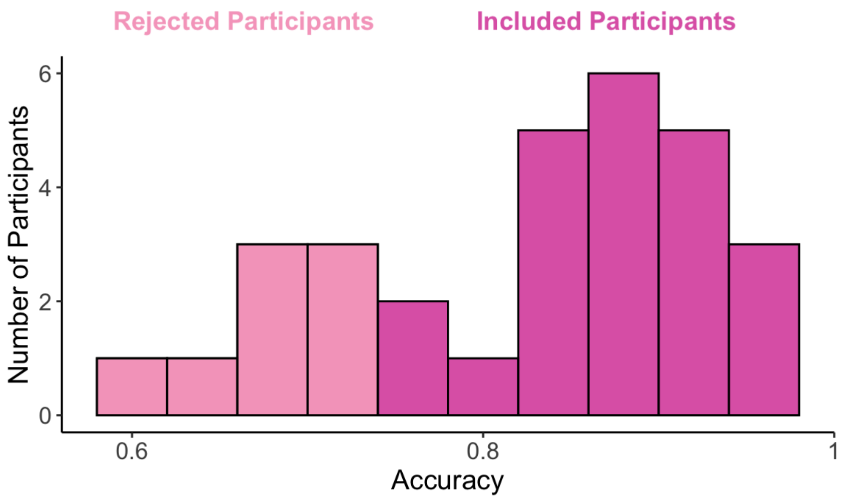

Supplementary Figure 1: Multiple-Choice Question Accuracy For Speech Rate ×1. The histogram shows the bimodal distribution of participants' mean multiple-choice accuracy at speech rate ×1. The x-axis shows mean accuracy; the y-axis shows the number of participants. Light pink indicates participants who failed the multiple-choice accuracy criterion; dark pink indicates those who passed.

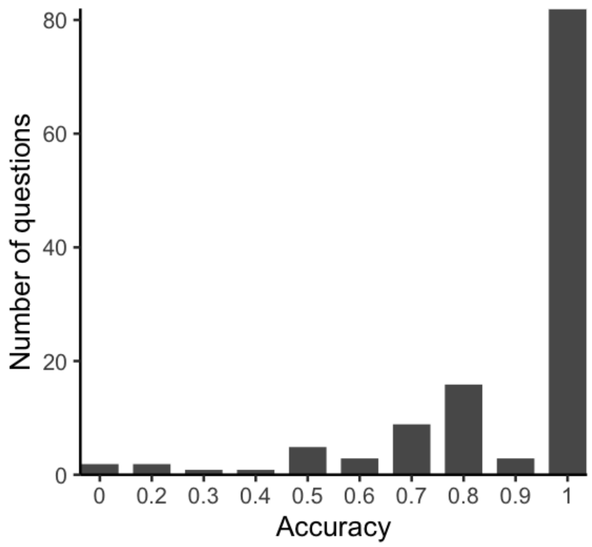

Supplementary Figure 2: Multiple-choice question accuracy per question across participants for speech rate x1. The figure shows multiple-choice question accuracy for each item in the non-sped-up condition among participants who passed the exclusion criterion. Most questions were answered at ceiling.

1.1.2 Replicating the results with all participants' data

We repeated the validation analyses including all participants (i.e., without the preregistered exclusions) to see if the main results would replicate. Single–predictor mixed-effects models predicting median slider scores from each post-hoc measure replicated: 10-point

scale, $\beta = 1.01$, $SE = 0.02$, $t(938.81) = 61.55$, $p < .001$; semantic similarity, $\beta = 1.33$, $SE = 0.08$, $t(477.57) = 17.29$, $p < .001$; multiple-choice accuracy, $\beta = 0.19$, $SE = 0.02$, $t(993.23) = 7.55$, $p < .001$. For these single–predictor models, Cohen's f^2 (all participants vs. included participants only) were: 10-point scale, 3.57 vs. 4.16; semantic similarity, 0.60 vs. 0.80; multiple choice, 0.05 vs. 0.14 (Supplementary Fig.3. Top Row).

In the model including all three post-hoc measures as predictors, the 10-point scale remained significant, $\beta = 1.04$, $SE = 0.04$, $t(458.01) = 26.54$, $p < .001$, whereas semantic similarity and multiple-choice accuracy did not explain additional independent variance (semantic similarity: $\beta = 0.05$, $SE = 0.07$, $t(400.61) = 0.74$, $p = .46$; multiple-choice: $\beta = 0.00$, $SE = 0.02$, $t(474.56) = -0.09$, $p = .93$). In this model, Cohen's f^2 (all participants vs. included participants only) were: 10-point scale, 1.32 vs. 1.57; semantic similarity, 0.00 vs. 0.15; multiple-choice, 0.00 vs. 0.00 (Supplementary Fig.3. Bottom Row).

Thus, all main results replicated, with the exception that semantic similarity no longer explained independent variance when all post-hoc measures were considered as predictors together. A likely explanation is that excluded participants wrote shorter summaries overall (mean words per summary: 14.13 vs. 18.99 for included participants) and at each speech-rate level (x1: 19.35 vs. 26.15; x2: 17.60 vs. 24.81; x3: 14.41 vs. 22.57; x4: 7.47 vs. 9.86; x5: 4.42 vs. 5.92).

Furthermore, the slider measure remained a highly significant predictor of speech rate even when we included participants who failed the preregistered checks ($\beta = -0.85$, $SE = 0.16$, $t(472.14) = -5.23$, $p < .001$.). This indicates that reduced performance stems from lack of engagement by some participants, not from a failure of the slider itself.

Finally, the recency analysis replicated when using the full sample. A Two-way ANOVA testing the main effects of speech rate and bins on semantic similarity scores showed that there is a main effect of speech rate but not of binning (speech rate: $F(1, 18746) = 3378.52$, $p < .001$; bin: $F(1, 18746) = 2.34$, $p = .13$), and the interaction was not significant ($F(1, 18746) = 0.14$, $p = .71$). As in the included-participants-only analysis, the measures that predicted comprehension less well (GloVe-Heard Segment and BERT)

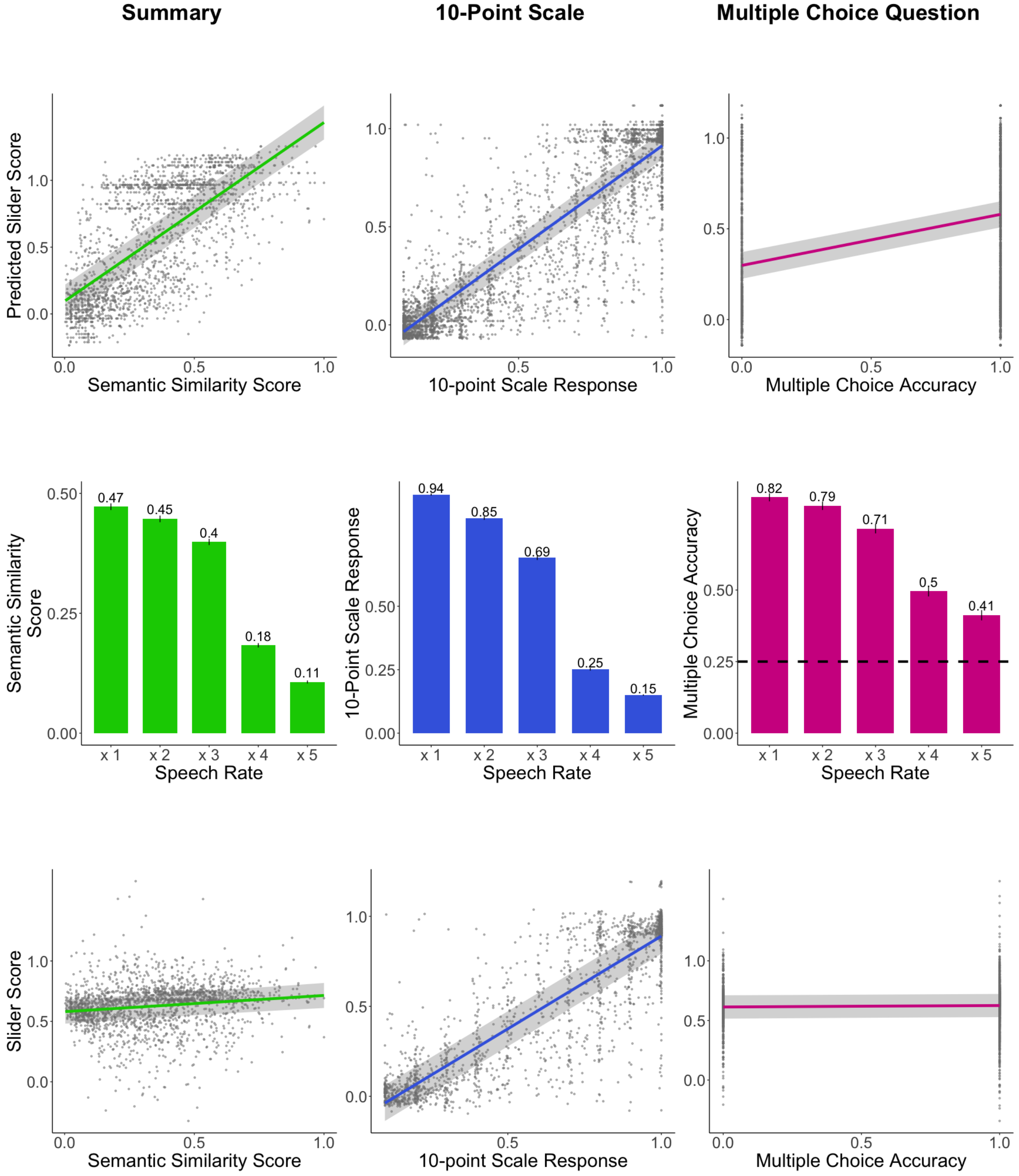

Supplementary Figure 3: Top row. Regression plots predicting slider scores from each post-hoc comprehension score. All post-hoc comprehension scores were a significant predictor of slider scores when utilized as the only fixed effect. Each plot shows the relationship between the specified post-hoc comprehension measure (x-axis) as the predictor and the predicted median slider score (y-axis). **Middle row.** Mean scores per speech rate for each post-hoc comprehension measure. Error bars represent standard error. The dashed line in panel in multiple choice accuracy plot represents the chance level. **Bottom row.** Regression predicting median slider scores from all post-hoc comprehension scores as fixed effects. Each plot shows the relationship between the specified post-hoc comprehension measure (x-axis) as the predictor and the predicted median slider score (y-axis), holding the other predictors constant at their mean values. Semantic similarity score was a significant predictor, whereas and 10-point scale ratings multiple choice question accuracy were not.

exhibited recency sensitivity, consistent with greater susceptibility to memory effects, whereas GloVe—Written Summary did not. Both GloVe—Heard Segment and BERT showed significant bin effects, with no interactions (GloVe—Heard Segment: speech rate $F(1, 9826) = 2549.42$, $p < .001$; bin $F(1, 9826) = 17.43$, $p < .001$; interaction $F(1, 9826) = 0.96$, $p = .33$; BERT: speech rate $F(1, 9826) = 806.94$, $p < .001$; bin $F(1, 9826) = 145.99$, $p < .001$; interaction $F(1, 9826) = 0.16$, $p = .69$).

1.2 Code for the slider software

MCU Pseudo-code:

1. Initialize hardware (timers, ADC, USB)
2. Open USB interface
3. Configure ADC to provide 8-bit values
4. In a loop, do:
    4.1. Record one slider value
    4.2. Record the time with another slider value
    4.3. Record another slider value
    4.4. Average the three slider values and send it along with the timestamp to the computer

The code for MCU would depend on the specific chip used for the design, here we are sharing a simplified version using Arduino: `https://github.com/irmak-ergin/measuring_naturalistic_speech_comprehension_2024/blob/main/experiment_1/experiment_files/slider_code_simple.ino`. This version is easier to implement and portable across devices. It reads slider values by checking the time and sleeping between samples. The version we used for data collection is more precise because it uses a hardware timer that automatically records data every millisecond, customized for a specific MCU. However, it produces the same type of output as the simplified Arduino version.

PsychoPy daemon pseudo-code:

1. Launch a new process where the next steps are performed. Note that it is essential this is a process and not a thread due to the Python Global Interpreter Lock.
2. Connect the USB interface to the MCU
3. For each trial, receive a message with the audio recording identifier of that trial
4. Keep track of the incoming slider values and timestamps during audio playback
5. Save the data and the clock drift

The code we used is available here: `https://github.com/irmak-ergin/measuring_naturalistic_speech_comprehension_2024/blob/main/experiment_1/experiment_files/v2.py`. One part runs in the background and continuously collects data from the slider. The other part defines the Slider element, which is added to the PsychoPy experiment to make the data available during each trial. Code snippets that enable real-time readouts for each trial are implemented directly in the PsychoPy experiment file: `https://github.com/irmak-ergin/measuring_naturalistic_speech_comprehension_2024/blob/main/experiment_1/experiment_files/experiment.psyexp`.

1.3 Correlation between speech comprehension measures

Here we report the correlation between all speech comprehension measures obtained in Experiment 1. Pearson correlations among the comprehension measures showed that slider scores were strongly associated with the 10-point scale responses (r = .90, $p <$ .001), and moderately associated with semantic similarity scores (r = .63, $p <$ .001) and multiple choice question accuracy (r = .35, $p <$ .001). Semantic similarity scores correlated with the 10-Point Scale (r = .65, $p <$ .001) and with multiple choice accuracy (r= .30, $p <$ .001). The 10-point scale also correlated with multiple choice question accuracy (r = .37, $p <$ .001) (Supplementary Fig.4).

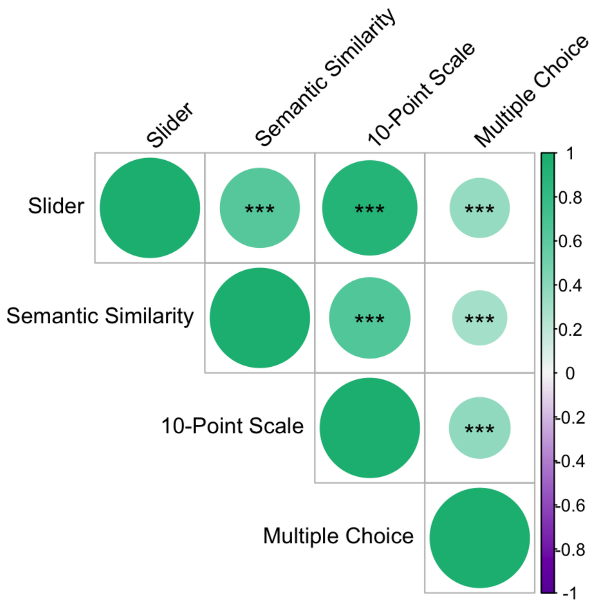

Supplementary Figure 4: Correlation Between Speech Comprehension Measures. ***= p<.001, **= p<.01, *= p<.05.

2 Experiment 2

2.1 By-participant variation in slider reports

As an attention check, we examined the distribution of slider movements across trials and participants, applying the same ±3.5 standard deviation criterion used in Experiment 1. All participants passed this criterion, including participants 6 and 8, although showing lower variability. It is also worth noting that the measure is able to capture individual differences in comprehension reports (Supplementary Fig. 5).

2.2 Using the slider with dominant vs non-dominant hand

Responses obtained with the dominant and non-dominant hand did not differ significantly (Supplementary Fig. 6).

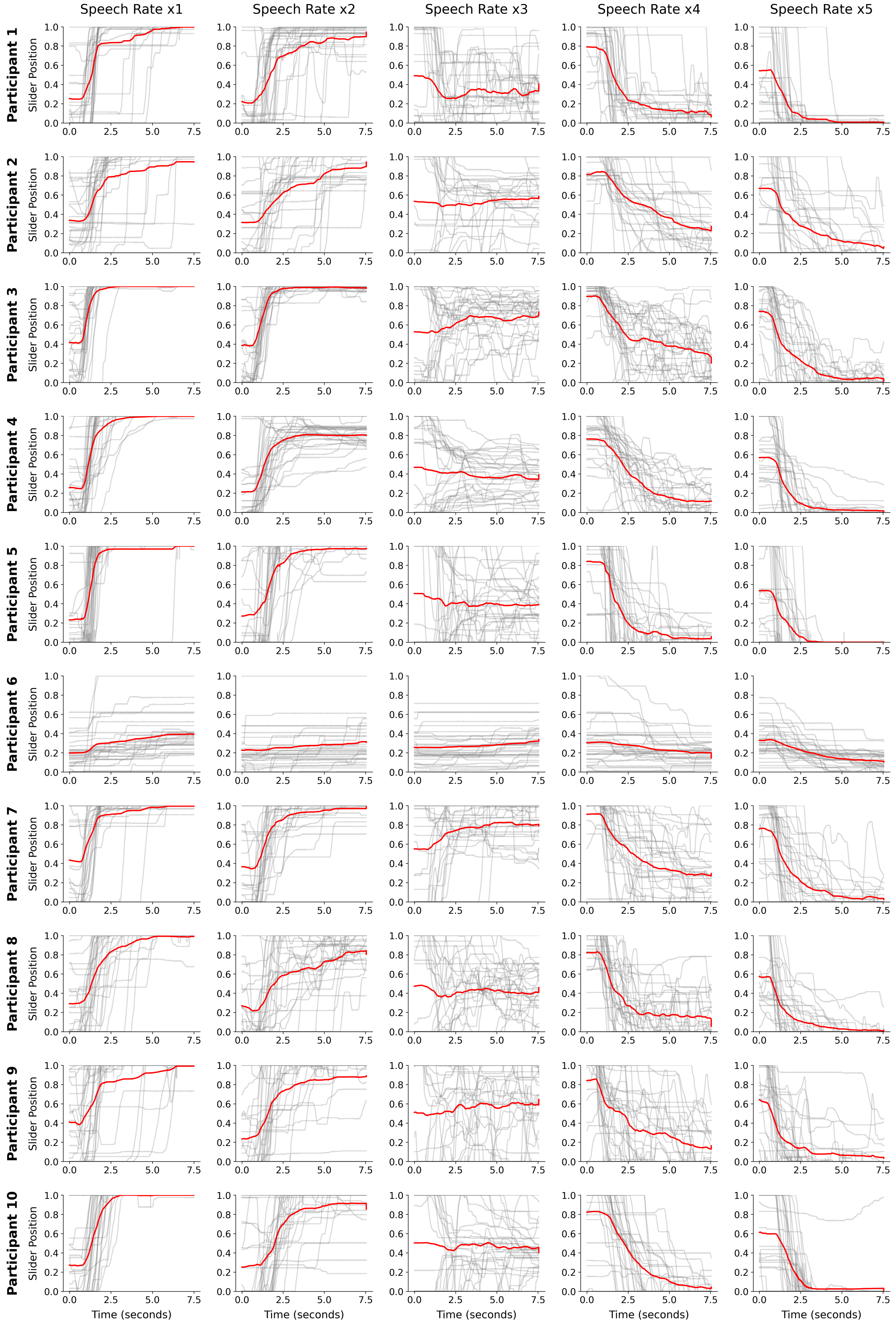

Supplementary Figure 5: Each participants slider scores. Normalized time-resolved slider scores reported via the slider during each trial for each speech rate. Grey lines represent individual trials, while red lines show the mean slider values across trials. The x-axis represents time in seconds, and the y-axis represents the slider position.

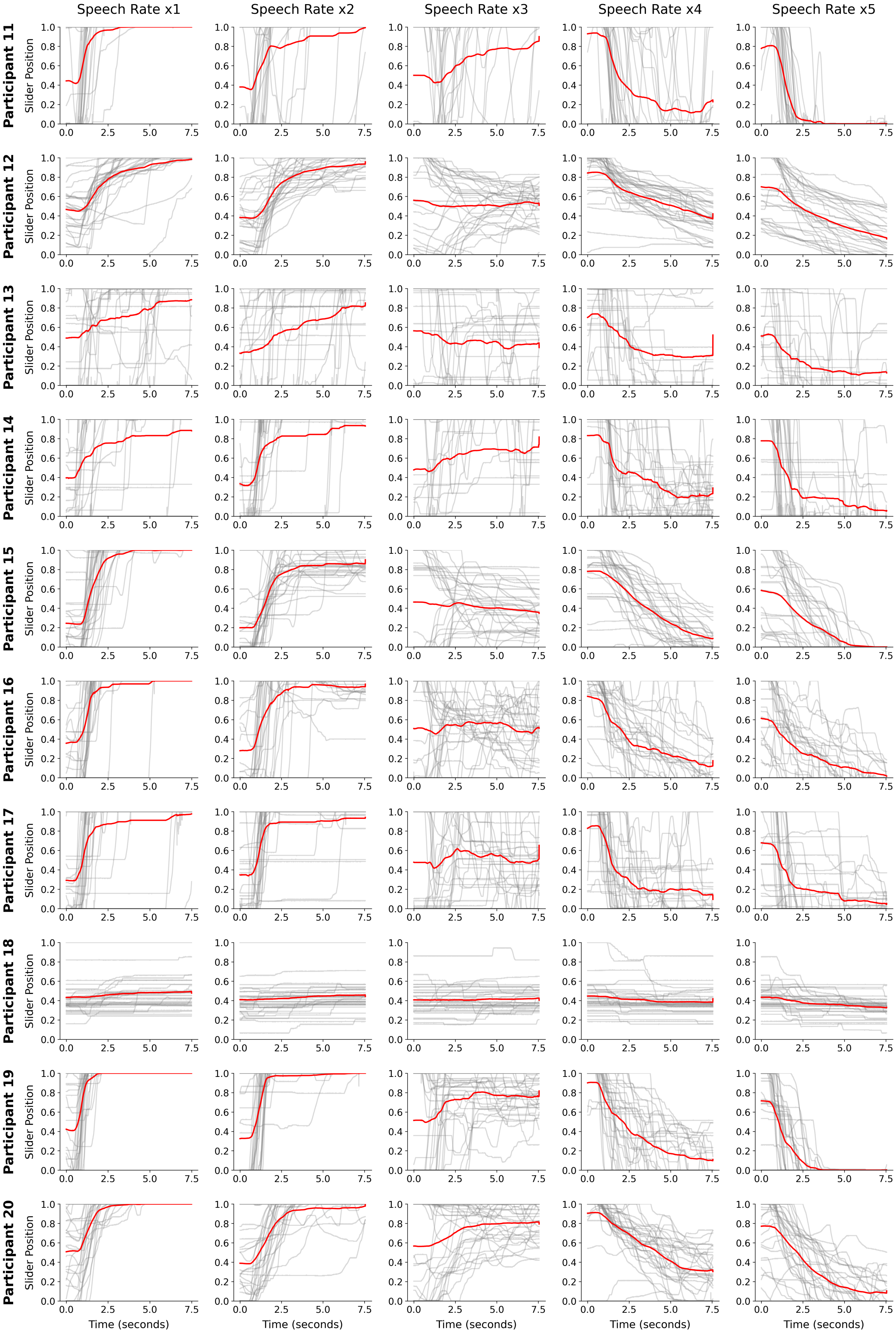

Supplementary Figure 5: Each participants slider scores. Normalized time-resolved slider scores reported via the slider during each trial for each speech rate. Grey lines represent individual trials, while red lines show the mean slider values across trials. The x-axis represents time in seconds, and the y-axis represents the slider position.

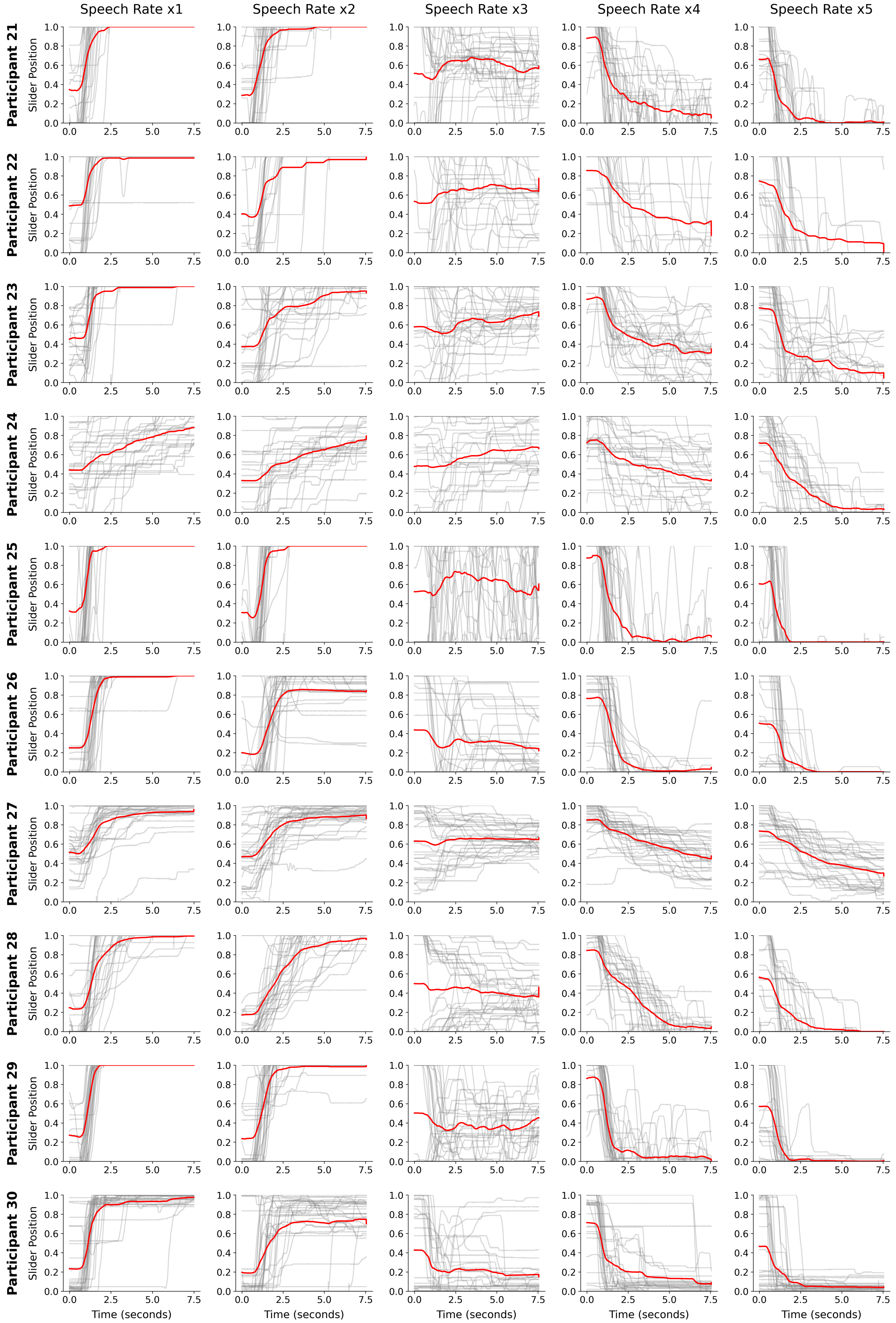

Supplementary Figure 5: Each participants slider scores Normalized time-resolved slider scores reported via the slider during each trial for each speech rate. Grey lines represent individual trials, while red lines show the mean slider values across trials. The x-axis represents time in seconds, and the y-axis represents the slider position.

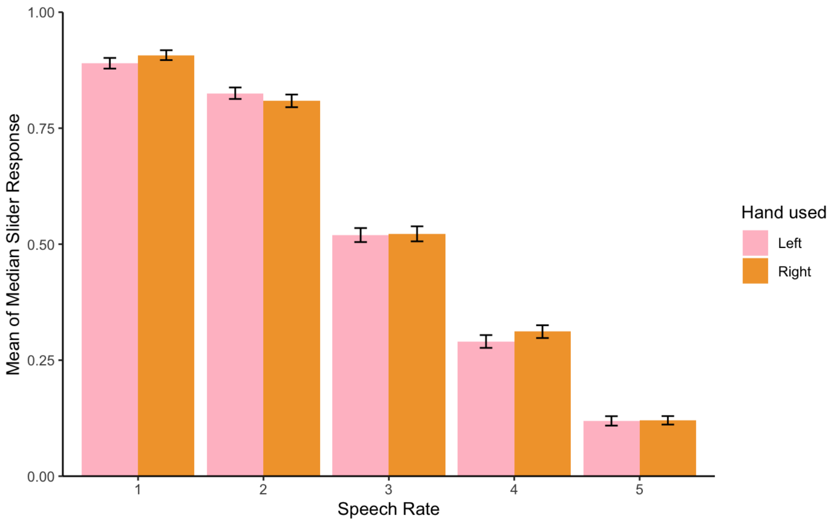

Supplementary Figure 6: Slider responses obtained via left vs. right hand.Pink bars represent slider responses reported using the left hand, orange bars represent slider responses reported using the right hand.

3 Experiment 3

3.1 Effects of speech rate presentation order on comprehension

As mentioned in the Design and Procedure section, we presented the same stimulus in different speech rates within participant to eliminate demand characteristic, unlike in Experiment 1. The choice of not doing this in experiment 1 was that we though hearing the same story for the second time can increase comprehension if they heard it in a slower speed before. To test whether multiple choice accuracy changed when participants heard the x1 version of the same segment first in comparison to when they heard the x2.5 version first, we fit a Binomial Generalized Linear Mixed–Effects Model predicting multiple choice accuracy from speech rate (x1 vs. x2.5), order (x1–first vs. x2.5–first), and their interaction. The main effects of speech rate and order were not significant (β_{rate} = -0.03, SE = 0.26, z = -0.13, p = .90; β_{order} = -0.03, SE = 0.26, z = -0.13, p = .90), but the speech rate–order interaction was significant ($\beta = -1.235$, SE = 0.339, z = -3.65, $p < .001$), indicating that the speed effect depended on which version was heard first. Estimated probabilities showed no difference when x1 came first ($\hat{p}_{\text{x1}}$ = 0.871, 95% CI

[0.817, 0.911]; $\hat{p}_{x2.5} = 0.868$, 95% CI [0.812, 0.908]; OR = 0.97, z = -0.13, p = .90), but a substantial drop when x2.5 came first ($\hat{p}_{x1} = 0.868$, 95% CI [0.812, 0.908]; $\hat{p}_{x2.5} = 0.648$, 95% CI [0.570, 0.719]; OR $\approx$ 0.28, $p < .001$).

Second, we tested whether this enhanced comprehension effect is present for slider scores, we fit a linear Mixed–Effects Model predicting median slider scores from speech rate (x1 vs. x2.5), order (x1-first vs. x2.5-first), and their interaction. There was a robust main effect of speech rate ($\beta = -0.322$, SE = 0.024, $t(506.20)$ = -13.61, $p < .001$), no main effect of order ($\beta = 0.002$, SE = 0.024, $t(505.86)$ = 0.09, p = .932), and a significant speech rate–order interaction ($\beta = -0.107$, SE = 0.034, $t(506.53)$ = -3.17, p = .002). Estimated marginal means showed higher scores at x1 than x2.5 when x1 was heard first ($\hat{M}_{x1} = 0.938$, 95% CI [0.818, 1.058]; $\hat{M}_{x2.5} = 0.616$, 95% CI [0.496, 0.736]), and a larger difference between x1 and x2.5 when x2.5 was heard first ($\hat{M}_{x1} = 0.940$, 95% CI [0.820, 1.061]; $\hat{M}_{x2.5} = 0.511$, 95% CI [0.391, 0.631]). Overall, as hypothesized, even though the multiple choice question items differed across the two presentations of the same segment, accuracy at x2.5 was higher when participants had previously heard that segment at x1; the same facilitation was evident in the slider scores (Fig.7).

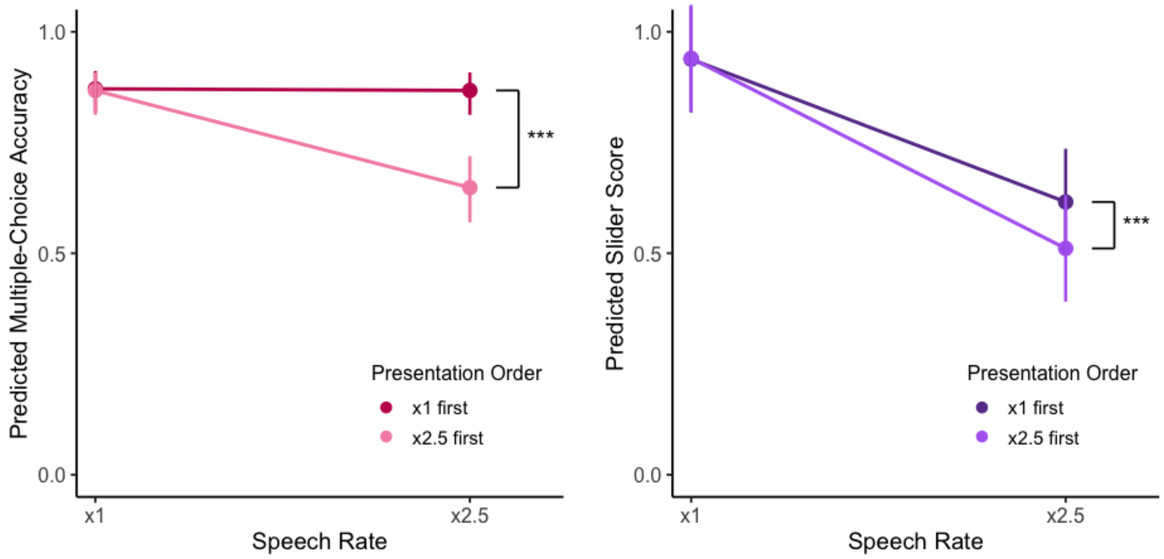

Supplementary Figure 7: Effects of speech-rate presentation order on comprehension.**Left panel:** Predicted multiple choice question accuracy from binominal general linear model with speech rate and presentation order as fixed effects. **Right panel:**Predicted sliders score from linear mixed-effects model with speech rate and presentation order as fixed effects. Points show estimated means; error bars denote 95% CIs. Brackets indicate order contrasts (x2.5 vs. x1); *** = p < .001. For both measures, comprehension was higher when participants heard the x1 version of a segment first compared to when they heard the x2.5 version first.

3.2 By-participant variation in TRFs

Below we show TRF models for each participant. As demonstrated in Experiment 1 and 2 (Fig. 5 & 13), and the device captures moment-by-moment comprehension and the variability across participants (Supplementary Fig. 5). Here, the TRF fits further show that the models can capture individual differences in response delays (Supplementary Fig. 8 shows actual and predicted slider positions by the TRF model for eahc participant, and Supplementary Fig. 9 shows the coefficients of each feature in the TRF model per participant.). Notably, Participant 22, despite meeting our exclusion criteria, exhibited an essentially flat TRF across features, suggesting very low variability in their slider responses and potentially reduced task engagement. Importantly, the slider makes such patterns visible. Future studies can leverage time-resolved responses and estimated response delays to implement stricter, data-informed exclusion procedures.

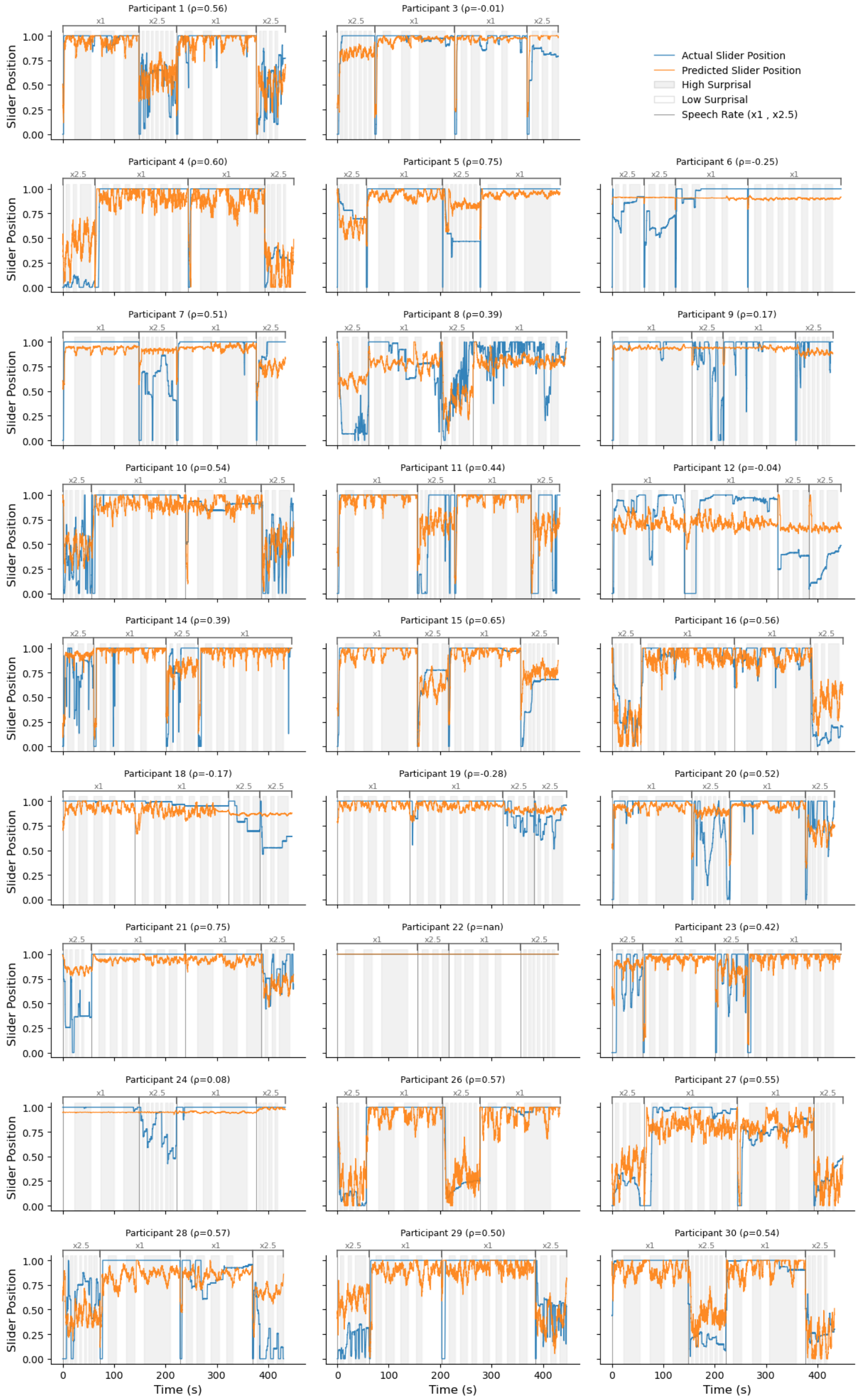

Supplementary Figure 8: Actual vs predicted slider responses for each participant. Each panel shows the actual slider position (blue) and the position predicted by the TRF model (orange) across the four stories in which the participants operated the slider while listening. Brackets above the traces indicate the speech rate for each story (x1 or x2.5). Shading within stories marks surprisal: white bands denote low-surprisal segments and gray bands denote high-surprisal segments.

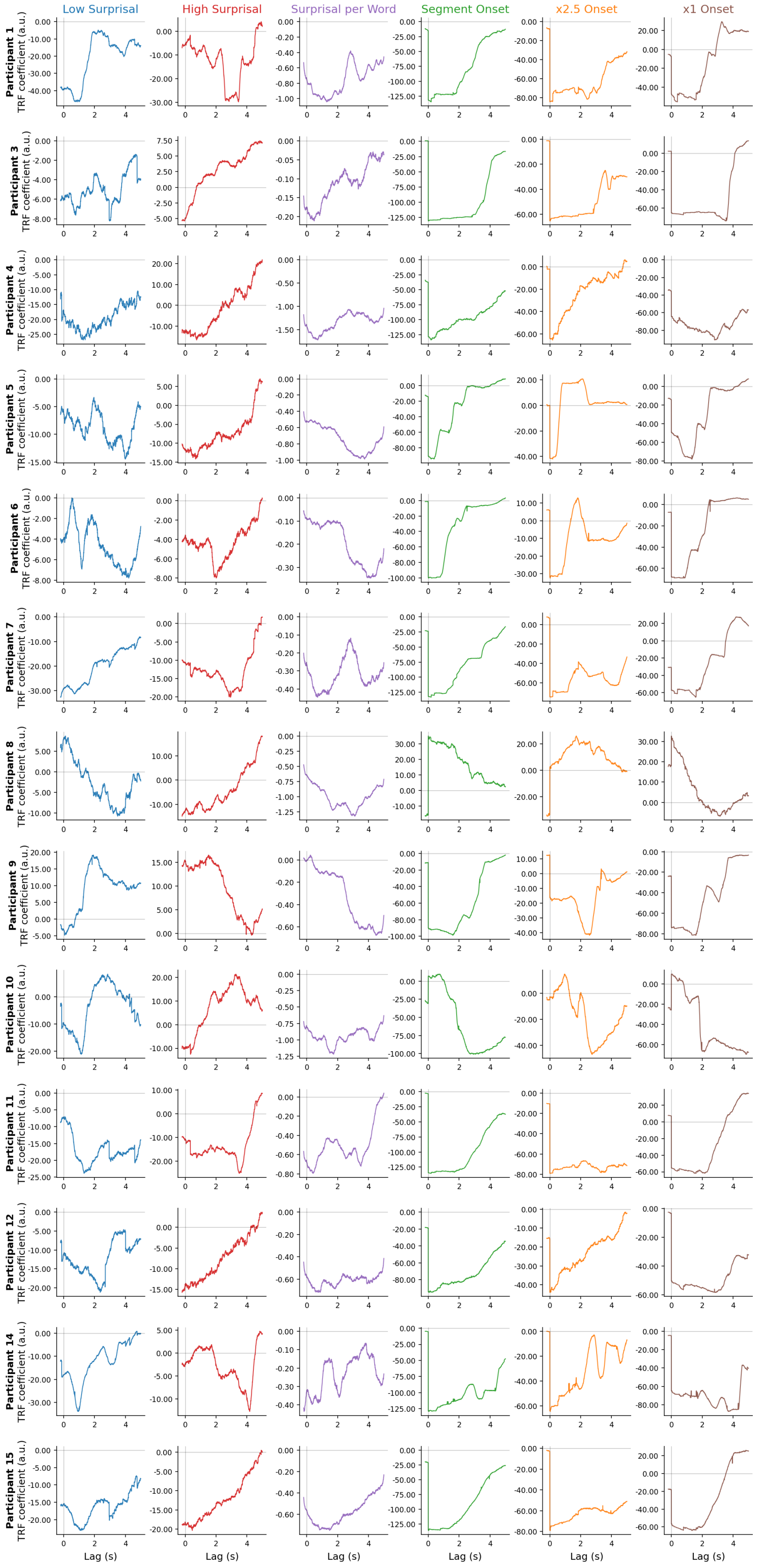

Supplementary Figure 9: TRF kernels of all features for each participant. Rows correspond to participants and columns to annotation features: (1) low surprisal chunk onsets, (2) high surprisal chunk onsets, (3) surprisal per word (each word's surprisal value), (4) segment onset, (5) onset of x1 speech rate segment , and (6) onset of x2.5 speech rate segment. The y axis shows the TRF coefficients (y axis) estimated over lags from -0.2 to 5 seconds (x-axis).

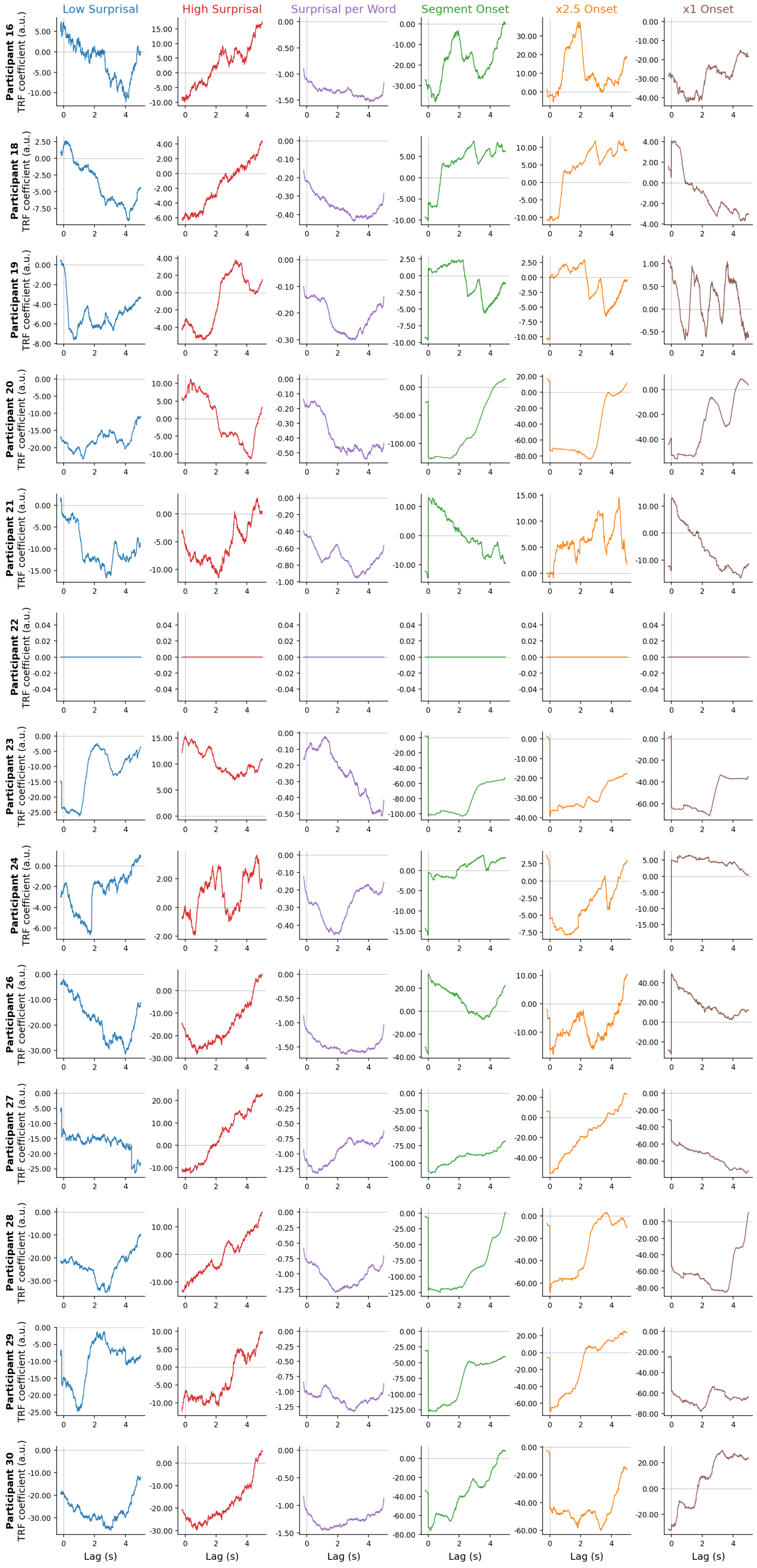

Supplementary Figure 9: TRF kernels of all features for each participant. Rows correspond to participants and columns to annotation features: (1) low surprisal chunk onsets, (2) high surprisal chunk onsets, (3) surprisal per word (each word's surprisal value), (4) segment onset, (5) onset of x1 speech rate segment , and (6) onset of x2.5 speech rate segment. The y axis shows the TRF coefficients (y axis) estimated over lags from -0.2 to 5 seconds (x-axis).
